# *N*‐Arachidonoyl‐phosphatidylethanolamide phospholipase D activation by muscarinic receptors

**DOI:** 10.1113/JP291508

**Published:** 2026-09-02

**Authors:** Natalia Murataeva, Connor Schmitt, Elyssa Hyman, Jim Wager‐Miller, Irene Matthews, Ruyi Cai, Shangxuan Cai, Yulong Li, Ken Mackie, Alex Straiker

**Affiliations:** ^1^ Department of Psychological and Brain Sciences Indiana University Bloomington Indiana USA; ^2^ Gill Institute for Neuroscience Indiana University Bloomington Indiana USA; ^3^ State Key Laboratory of Membrane Biology Peking University School of Life Sciences Beijing China; ^4^ PKU‐IDG/McGovern Institute for Brain Research Beijing China; ^5^ Peking‐Tsinghua Center for Life Sciences, New Cornerstone Science Laboratory Academy for Advanced Interdisciplinary Studies Beijing China

**Keywords:** acylethanolamine, anandamide, endocannabinoid, *N*‐arachidonoyl‐phosphatidylethanolamide phospholipase D (NAPE‐PLD)

## Abstract

**Abstract:**

The endogenous cannabinoid signalling system consists of G protein‐coupled receptors, messengers – 2‐arachidonoylglycerol (2‐AG) and anandamide – and enzymatic machinery to synthesize and metabolize these messengers. Anandamide is physiologically important, but its synthesis is incompletely understood. *N*‐Arachidonoyl‐phosphatidylethanolamide phospholipase D (NAPE‐PLD) synthesizes acylethanolamines, including anandamide, but how is NAPE‐PLD activated? We used genetically encoded G protein‐coupled receptor based (GRAB) sensors for endocannabinoids (eCBs) and immunohistochemistry to investigate. Human embryonic kidney 293 (HEK293) cells natively express G_q_‐coupled muscarinic M_3_ receptors. The muscarinic agonist oxotremorine‐M stimulated the GRAB_eCB_ sensor only when HEK293 cells were cotransfected with NAPE‐PLD. This signal was reduced by the NAPE‐PLD inhibitor LEI401 and required both phospholipase C and internal calcium stores. G_q_‐coupled mGluR5 glutamate receptors effectively substitute for M_3_ receptors. Thus M_3_ receptors stimulate NAPE‐PLD synthesis of acylethanolamines such as anandamide via G_q_ signalling pathways resembling those for 2‐arachidonoylglycerol. Parasympathetic activation stimulates tearing and salivation via M_3_ receptors on myoepithelial cells. The cannabinoid signalling system may act as a feedback inhibitor to inhibit acetylcholine release, but the identity and source of the endogenous cannabinoid messenger are uncertain. NAPE‐PLD and M_3_ proteins colocalize in myoepithelial cells; fatty acid amide hydrolase (FAAH) resides in glandular acinar cells. Oxotremorine‐M stimulation of lacrimal or submandibular salivary glands co‐cultured with HEK293‐GRAB_eCB_ cells stimulated GRAB_eCB_ responses. This response was diminished by LEI401 and was also present in cells expressing GRAB_AEA_, an anandamide‐specific sensor, but not those expressing GRAB_2AG_. We conclude that muscarinic M_3_ receptors stimulate NAPE‐PLD to produce anandamide in cell lines and exocrine glands. This means of stimulating anandamide synthesis may play many roles in the body.

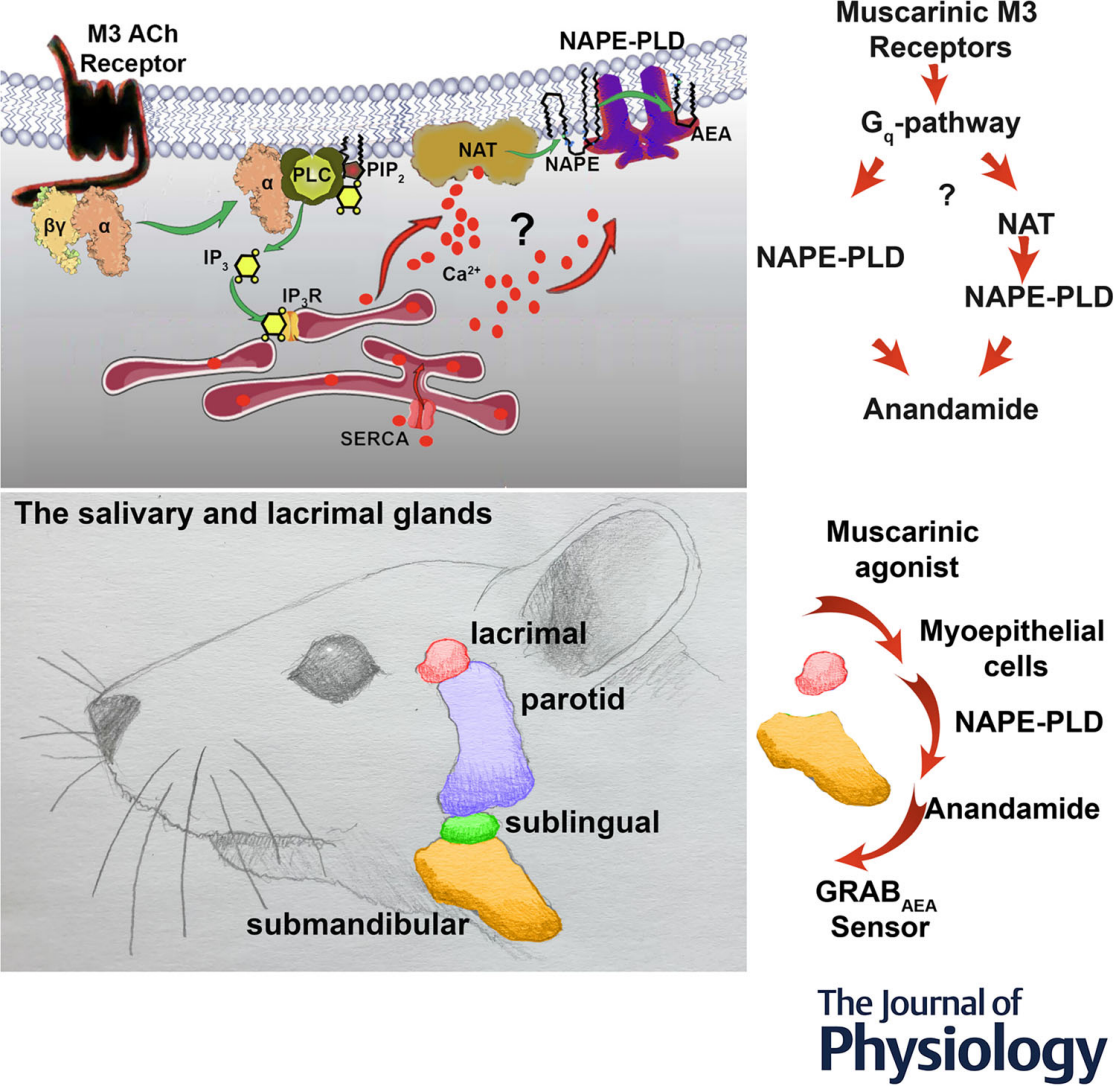

**Key points:**

- The cannabinoid signalling system plays important roles in the body and in doing so makes use of two endogenous messengers, 2‐arachidonoylglycerol (2‐AG) and anandamide. The regulation of anandamide synthesis is still poorly understood.
- This study demonstrates that muscarinic G_q_‐coupled GPCRs can stimulate anandamide production by activating the enzyme *N*‐arachidonoyl‐phosphatidylethanolamide phospholipase D (NAPE‐PLD) and further shows that this occurs natively in salivary and lacrimal glands.
- Muscarinic G_q_‐coupled G protein‐coupled receptor (GPCR) activation of NAPE‐PLD to induce anandamide synthesis may play many roles in the body.

## Graphical abstract

**Abstract figure legend** Activating muscarinic M_3_ receptors activates G_q_‐pathway signalling, including the activation of phospholipase C (PLC) and release of calcium from internal stores as shown in the upper panel. It remains unclear whether the calcium acts on *N*‐arachidonoyl‐phosphatidylethanolamide phospholipase D (NAPE‐PLD) itself or upstream at *N* acyl transferase (NAT). In lacrimal and submandibular salivary glands of mice, stimulation of muscarinic receptors by oxotremorine‐M activates NAPE‐PLD‐dependent production of anandamide.

## Introduction

The endogenous cannabinoid signalling system consists of G protein‐coupled receptors, messengers – 2‐arachidonoylglycerol (2‐AG) and arachidonoylethanolamide (AEA, anandamide) – and the enzymatic machinery to synthesize and metabolize these messengers. Anandamide activates cannabinoid receptors (CB1 and CB2; Devane et al., 1992; Munro et al., 1993) but also transient receptor potential vanilloids 1 (TRPV1) (Smart et al., 2000) and may play many roles in the body. Reducing anandamide metabolism by blocking fatty acid amide hydrolase (FAAH) increases anandamide levels (Cravatt et al., 2001). Anandamide is implicated in many important functions, including pain (Cravatt et al., 2001; Jayamanne et al., 2006) and salivation (Andreis et al., 2022). Though anandamide has important physiological roles, our understanding of its synthesis has lagged behind that of 2‐AG. It is now appreciated that anandamide and related acylethanolamines are often produced enzymatically by *N*‐acyl phosphatidylethanolamine phospholipase D (NAPE‐PLD), a zinc metallohydrolase (Daiyasu et al., 2001; Devane et al., 1992; Schmid et al., 1983). Mice with NAPE‐PLD genetically deleted have lower levels of several acylethanolamines, including anandamide (Leishman et al., 2016). NAPE‐PLD has binding sites for both zinc and bile salts (Magotti et al., 2015) that modulate NAPE‐PLD function. However our understanding of how NAPE activity and AEA synthesis are regulated remains limited. Early work demonstrated that neuronal depolarization can induce anandamide production in a calcium‐dependent manner, perhaps via the activation of *N*‐acyl transferase (NAT) that then provided the NAPE substrate for NAPE‐PLD (Di Marzo et al., 1994). However a study with purified NAPE‐PLD showed that the enzyme can be activated by Ca^2+^ (Ueda et al., 2001). Evidence that an intracellular calcium increase is necessary for some forms of anandamide production (Ligresti et al., 2004) led to the proposition that G_q_‐coupled receptors may directly or indirectly induce the synthesis of anandamide similar to the way that the diacylglycerol lipase (DAGL) enzymes can be activated to produce 2‐AG (Bisogno et al., 2003; Jain et al., 2013; Straiker & Mackie, 2007; van der Stelt & Di Marzo, 2005).

**Figure 1:**
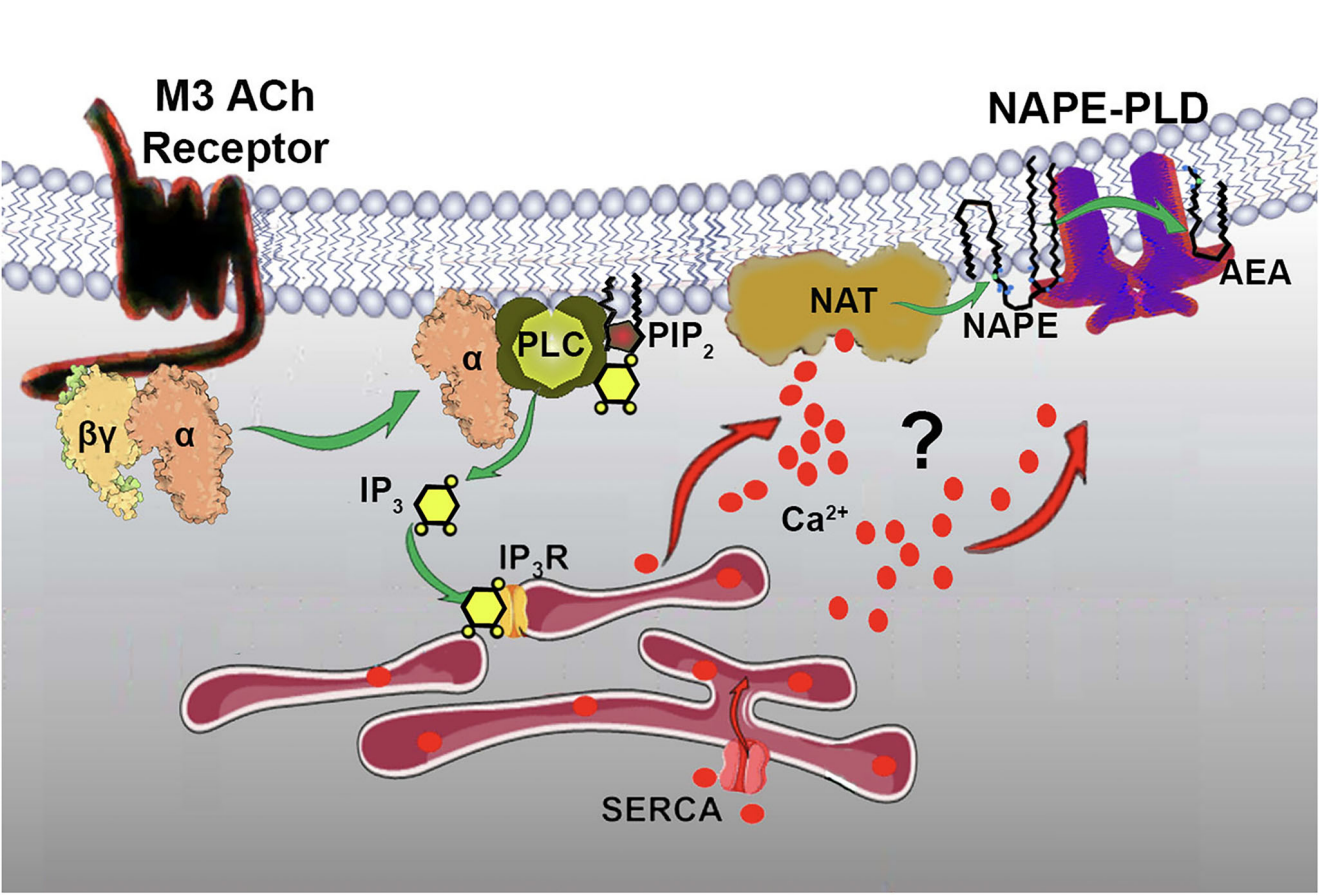
Schematic of proposed muscarinic activation of NAPE‐PLD (*N*‐arachidonoyl‐phosphatidylethanolamide phospholipase D) Proposed mechanism of action involves muscarinic M_3_ acylcholine (ACh) receptor activation. The Gα subunit activated by(muscarinic M_3_ receptor) M_3_R stimulates phospholipase C (PLC) to cleave IP_3_ from (phosphatidylinositol 4,5‐bisphosphate) PIP_2_. IP_3_ activates IP_3_ receptors on internal calcium stores, releasing calcium. Calcium acts directly on *N*‐acyl transferase (NAT) which then produces the NAPE precursor that is then acted upon by NAPE‐PLD to produce anandamide (AEA (arachidonoylethanolamide)). Calcium may also directly activate NAPE‐PLD.

The recently developed suite of endocannabinoid (eCB) sensors offers an opportunity to probe in depth the role of NAPE‐PLD in anandamide production in real time in intact cells and tissues. GRAB endocannabinoid sensors (e.g. GRAB_eCB_) are modified CB1 receptors incorporating a circularized green fluorescent protein (GFP) that fluoresces in response to a ligand binding to the CB1 receptor (Dong et al., 2022). A general GRAB_eCB_ sensor recognizing anandamide and 2‐AG has been characterized in cell lines (Singh et al., 2023) as well as in *ex vivo* and *in vivo* preparations (Dong et al., 2022). Muscarinic M_3_ receptors are G_q_‐coupled GPCRs natively expressed in HEK293 cells (Atwood et al., 2011). Activating G_q_‐coupled receptors has been shown to induce the synthesis of 2‐AG by DAGLs (Bisogno et al., 2003; Jain et al., 2013; Straiker & Mackie, 2007). We have recently demonstrated that co‐expressing this sensor with a 2‐AG‐synthesizing enzyme, DAGLα, permits a real‐time readout of 2‐AG production stimulated by muscarinic M_3_ receptors (Dvorakova et al., 2025). We hypothesized that the activation of natively expressed M_3_ receptors might similarly activate the anandamide‐synthesizing machinery to stimulate the production of acylethanolamines, including anandamide. Figure 1 shows the proposed signalling pathways involved in M_3_ activation of NAPE‐PLD.

The eCB system is well positioned to regulated tearing, salivation and other exocrine gland function. Activation of muscarinic M_3_ receptors by acylcholine (ACh) released from parasympathetic fibres stimulates tearing and salivation (reviewed in Mitchelson, 2012). We have found that CB1 cannabinoid receptor activation inhibits tearing as well as basal and stimulated salivation from lacrimal and salivary glands (Andreis et al., 2022; Murataeva, Mattox et al., 2024; Thayer et al., 2020). These findings likely explain the frequent reports of dry mouth and dry eye in cannabis users. CB1 receptors are located on the axons of parasympathetic inputs where they are well placed to inhibit ACh release. Thus G_q_‐stimulated synthesis of eCBs in lacrimal and salivary glands is an attractive mechanism to negatively regulate parasympathetic ACh release. However the identity and source of the endogenous cannabinoid messenger are unknown.

Here using GRAB_eCB_ sensors we first investigated the muscarinic stimulation of anandamide synthesis in a NAPE‐PLD‐transfected cell line that natively expresses muscarinic M_3_ receptors. Then using immunohistochemistry we localized FAAH and NAPE‐PLD protein expression in salivary and lacrimal glands. Finally we made use of GRAB_eCB_ and anandamide/2‐AG‐selective GRAB sensors to detect eCB production stimulated by natively expressed M_3_ receptors in these glands.

## Methods

### Animals

Lacrimal and submandibular salivary glands (SMGs) were obtained from mice that were housed in standard ventilated caging (cage floor dimensions: 33.5 ×18 cm, depth 14 cm), with corn cob‐based bedding (Bed‐o'cobs Laboratory Animal Bedding, The Andersons, Maumee, OH, USA). Mice were group housed three to four per cage and were fed Inotiv Teklad 2918 irradiated rodent diet *ad libitum*. Adult mice (both sexes, age 2–4 months) were used as gland donors for functional experiments, but see Preparation of lacrimal and submandibular glands section below regarding salivary glands. Because mice are nocturnal we collected the glands during their active phase by maintaining them on a reverse light cycle (lights turned on 9:00 PM to 9:00 AM). All animal care and experimental procedures used in this study were approved by the Institutional Animal Care and Use Committee of Indiana University (protocol no. 24‐030, approval date 3 September 2024) and conformed to the Guidelines of the National Institutes of Health on the Care and Use of Laboratory Animals. Experiments complied with ARRIVE guidelines. Anaesthesia was required only for killing the animals to obtain tissues as described in the ‘Immunohistochemistry’ section.

### A cannabinoid sensor‐based assay of eCB synthesis

**Figure 2:**
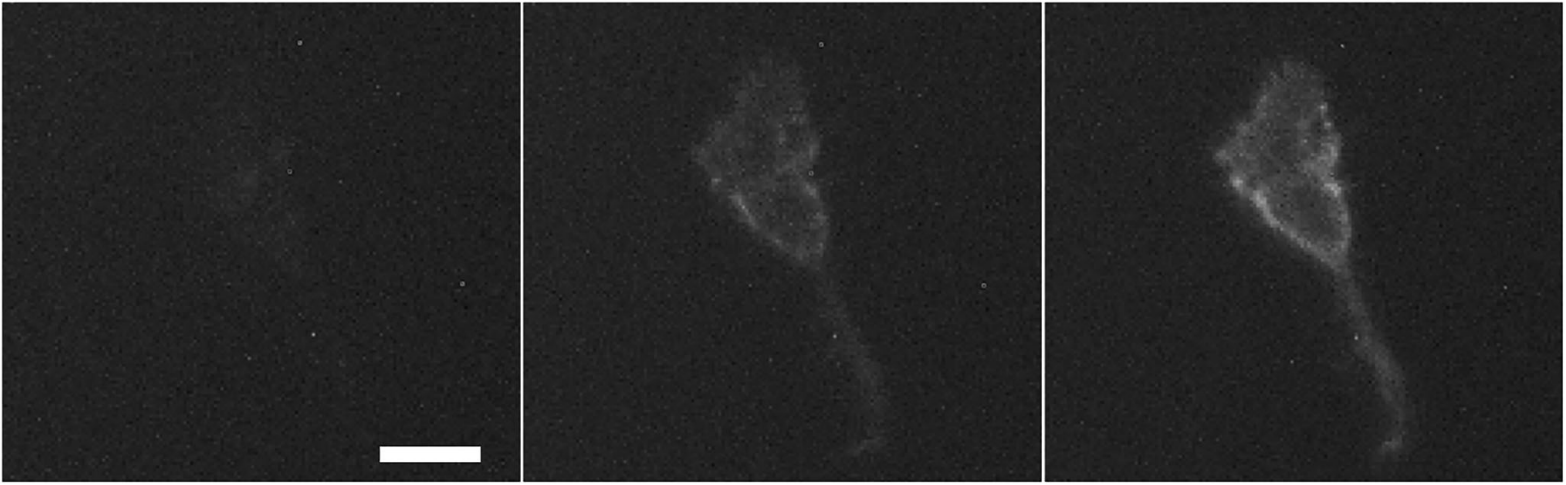
Sample HEK‐GRAB_eCB3.0_ fluorescence Sequence of fluorescence responses in a single HEK293 cell expressing GRAB_eCB3.0_ and NAPE‐PLD (*N*‐arachidonoyl‐phosphatidylethanolamide phospholipase D). The images show baseline (left), oxo‐M (oxotremorine‐M)‐stimulated (centre) and 2‐AG (2‐arachidonoylglycerol)‐stimulated response (right). In experiments responses are taken from ∼6 to 9 cells (technical replicates) and averaged to serve as a data point. Scale bar: 5 µm.

HEK293 cells were co‐transfected with NAPE‐PLD and the GRAB_eCB_ sensor (Dong et al., 2022; Singh et al., 2023) or the GRAB_eCB_ sensor alone using Lipofectamine 3000 according to the manufacturer's instructions (Thermo Fisher Scientific, Waltham MA). We used GRAB_eCB_, version 3.0, for these experiments (Dvorakova et al., 2025). For some experiments we used sensors developed to be selective for anandamide (GRAB_AEA_) or 2‐AG (GRAB_2AG_) (Cai et al., 2024). After transfection was visually confirmed using fluorescence, cells were transferred to 48‐well plates and tested the following day. Transfected cells were visualized in a given well and recorded using an inverted Nikon Instruments microscope fitted with a Spectra‐X light engine (Lumencor, Beaverton, OR, USA), a Flash 4 Hamamatsu camera (Hamamatsu City, Japan) and Nikon Elements AR software. At the end of each experiment, cells were treated with 10 µM 2‐AG or 30 µM anandamide to determine a maximal response (see Fig. 2 for sample fluorescence responses). These concentrations were determined experimentally. Oxotremorine‐M (oxo‐M) responses were evaluated as a percentage of the maximal 2‐AG or anandamide response. Multiple cells (6–9) in a well were averaged for a single experiment (technical replicates). Multiple wells were tested (biological replicates) from at least two separate batches on separate days. Before a given set of experiments, cell media was replaced with Earle's Balanced Salt Solution (EBSS, ThermoFisher Scientific, Waltham, MA, USA). Successive drug treatments were added directly to a given well at 2×, 3× and 4× concentrations as appropriate. Bovine serum albumen (BSA) has been reported to increase signals in experiments using the GRAB_eCB_ sensor (Singh et al., 2023), presumably by serving as a carrier for lipophilic eCBs. The initial experiments primarily involved the production and detection of eCBs in the same cell, so BSA was not added as we have not found BSA to be necessary to observe physiological response after bath application of eCBs (e.g. Straiker & Mackie, 2005). However the experiments using exocrine glands postulate that myoepithelial cells produce eCBs that are then released into the medium. In this circumstance the inclusion of BSA might facilitate the release of eCBs into the media and transport to the HEK293 cells that serve as a sensor. When experiments were performed in the close confines of a well in a 48‐well plate, BSA was not included, but in the larger volume of a Petri dish with a 25 mm cover glass bottom, we did not observe an oxo‐M response unless 0.2% BSA was added.

### Preparation of lacrimal and submandibular glands

For gland collection mice were killed using isoflurane (5%) followed by cervical dislocation. In the case of rat tissue, a rat was killed using isoflurane (5%) followed by decapitation as a secondary method. SMGs were collected from either sex, whereas for external lacrimal glands (ELGs), SMGs were collected only from female mice. We did not observe sex dependence of cannabinoid regulation of salivation (Andreis et al., 2022). However cannabinoid regulation of tearing occurs with a circadian regulation only in female mice (Murataeva, Yust et al., 2024). Moreover women suffer disproportionately from dry eye and dry mouth; for instance 80% of those suffering from Sjögren's syndrome, an autoimmune disease that causes dry eye and dry mouth, are female (Brandt et al., 2015). The head was removed from the body by decapitation at the C7/T1 junction to ensure no damage to the SMG occurred. Once the head was separated the SMG was excised and transferred to a large Petri dish (100 mm) with chilled PBS. In the dish the extraneous tissue, such as the adjoining adipose and lymphatics, was removed, and the glands were separated into left and right SMGs and placed in fresh, cold PBS‐filled small Petri dishes (35 mm). The ELG was also obtained from the same mouse head. Similarly the ELG was first placed in a large Petri dish with cold PBS, and extraneous tissue was trimmed away. The ELG was then transferred to a new, small Petri dish containing cold PBS. To prepare gland slices each gland was placed in a new, small Petri dish filled with cold PBS. The capsule of each gland was removed, and the gland then was manually sliced into three sections of equal size. Each section was used within 3 h of dissection to ensure optimal tissue condition.

### Immunohistochemistry

For immunohistochemistry lacrimal glands were fixed in 4% paraformaldehyde for 45 min at 4°C, and then placed sequentially in 10% and 30% sucrose in PBS overnight before being suspended in OCT (ThermoFisher Scientific, Waltham MA, USA) freezing compound in a 15 mL plastic test tube, to allow precise orientation in three‐dimensional space. The tube was then promptly submerged in cold (–80°C) ethanol to rapidly freeze the sample. Fixed lacrimal tissues were sectioned at 35 µm thickness on a Leica cryostat (Leica Microsystems, Wetzlar, Germany), and sections were mounted on Superfrost Plus slides (ThermoFisher). Slides were blocked with BSA (in PBS, Triton X, 0.3%) and then treated with primary antibodies (in PBS, Triton‐X, 0.3%) for 1–2 days at 4°C. See Table 1 for a list of primary antibodies. Where secondary antibodies were required a second incubation with secondary antibody (∼4 h at RT) was performed after the primary antibody was washed off. Phalloidin488/594 was included with secondary antibodies as appropriate to visualize acinar cells. Secondary antibodies were labelled with Alexa488, Alexa594 or Alexa647 (ThermoFisher) as appropriate for the experiment. Slides were mounted with mounting media containing 4',6‐diamidine‐2'‐phenylindole dihydrochloride (DAPI) to visualize nuclei (Fluoromount, Sigma‐Aldrich, St. Louis, MO, USA). Images were obtained using a Leica TCS SP8 (Leica Microsystems). Images were processed using FIJI (available at https://imagej.net/Fiji/downloads) and/or Photoshop (Adobe Inc., San Jose, CA, USA) software. Images were modified only in terms of brightness and contrast.

**Table 1:** Primary antibodies/labels used in this study

Target	Host	Source	Category number	Lot number	RRID	Concentration
FAAH	Rabbit	Synaptic Systems	469003	NA	RRID:AB_2924950	1:300
NAPE‐PLD	Guinea‐pig	Frontier Institute	NAPE‐PLD‐GP‐Af720	NA	RRID:AB_2571806	1:300
Muscarinic M_3_ receptor	Rabbit	Alomone	AMR006	AN0625	RRID:AB_2039997	1:300
Smooth muscle actin	Mouse	Sigma	A5228	056M4828V	RRID:AB_262054	1:300
Phalloidin‐488, 594	NA	ThermoFisher	A12379	1749905	NA	1:500

FAAH, fatty acid amide hydrolase; NAPE‐PLD, *N*‐arachidonoyl‐phosphatidylethanolamide phospholipase D; NA, not applicable.

### Drugs

Anandamide, 2‐AG, oxo‐M, edelfosine, thapsigargin, URB597, oleoylethanolamide (OEA), palmitoylethanolamide (PEA), linoleoylethanolamide (LEA) and LEI401 were purchased from Cayman Chemical (Ann Arbor, MI, USA). Cannabidiol (CBD) was obtained through the NIDA Drug Supply programme ADL‐19496‐11.

### Quantification and statistical analysis

GraphPad Prism, version 10 (La Jolla, CA, USA), software was used for statistical analysis. Data are presented in the figures as means ± SD. Means ± SD and *n* values are provided in the text. The statistical details of experiments can be found in the ‘Results’ section. Differences were considered statistically significant at *P* ≤ 0.05.

## Results

### The muscarinic agonist oxo‐M activates eCB sensor response in HEK293 cells expressing NAPE‐PLD and GRAB_eCB_

**Figure 3:**
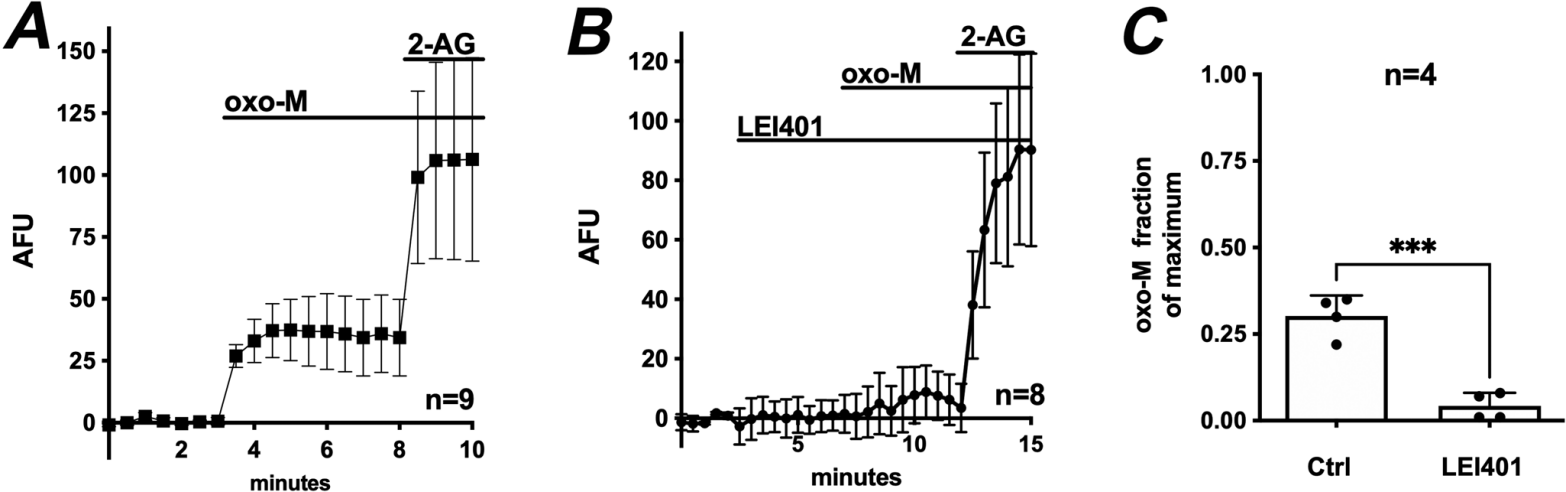
The muscarinic agonist oxotremorine‐M (oxo‐M) activates an endocannabinoid (eCB) sensor response in HEK293 cells expressing NAPE‐PLD (*N*‐arachidonoyl‐phosphatidylethanolamide phospholipase D) and GRAB_eCB_ *A*, sample time course from a single experiment showing GRAB_eCB_ responses to oxo‐M (10 µM) followed by the CB1 receptor agonist 2‐arachidonoylglycerol (2‐AG, 10 µM). *B*, sample time course averaged from a single experiment showing responses to oxo‐M and 2‐AG after pre‐treatment with NAPE‐PLD inhibitor LEI‐401 (3 µM, 5 min). *C*, bar graph showing LEI401 inhibition of oxo‐M responses as a fraction of 2‐AG effects in same‐day experiments. Values taken 2 min after oxo‐M treatment. ^***^ *P* = 0.0003, unpaired *t* test *versus* control. Each data point in panel C represents a pooled response from 5 to 9 cells. Data in figures are presented as values (±SD).

As noted HEK293 cells natively express muscarinic M_3_ receptors (Atwood et al., 2011). In cells transfected with both the GRAB_eCB_ sensor (version 3.0) and NAPE‐PLD, the muscarinic agonist oxo‐M (10 µM) stimulated a GRAB_eCB_ sensor response (Fig. 3*A,C*; response to oxo‐M as a fraction of 2‐AG (10 µM) response (± SD): 0.287 ± 0.091, *n* = 4). We have shown previously that this oxo‐M response is absent in cells transfected only with the eCB sensor (Dvorakova et al., 2025). Across many experiments the oxo‐M response was generally 25%–35% of the maximal sensor response as determined by subsequent activation by 2‐AG (10 µM). We chose 2‐AG for these initial experiments because it has worked well with this sensor in our previous study (Dvorakova et al., 2025). In side‐by‐side treatments AEA and 2‐AG similarly activated the GRAB_eCB3.0_ sensor (30 µM AEA response as a fraction of equimolar 2‐AG response (±SD): 0.697 ± 0.129, *n* = 3, data not shown). Pre‐treatment with the NAPE‐PLD inhibitor LEI401 (Mock et al., 2020; 3 µM, 5 min) prevented the response (Fig. 3*B,C*, control oxo‐M response as a fraction of maximal activation (±SD): 0.303 ± 0.059, *n* = 4; LEI401: 0.0425 ± 0.0378, *n* = 4; ***P = 0.0003 using unpaired *t* test *vs*. control, *t* = 7.3, df = 6, *n* = 4).

### Muscarinic activation of NAPE‐PLD requires PLC and full internal calcium stores

**Figure 4:**
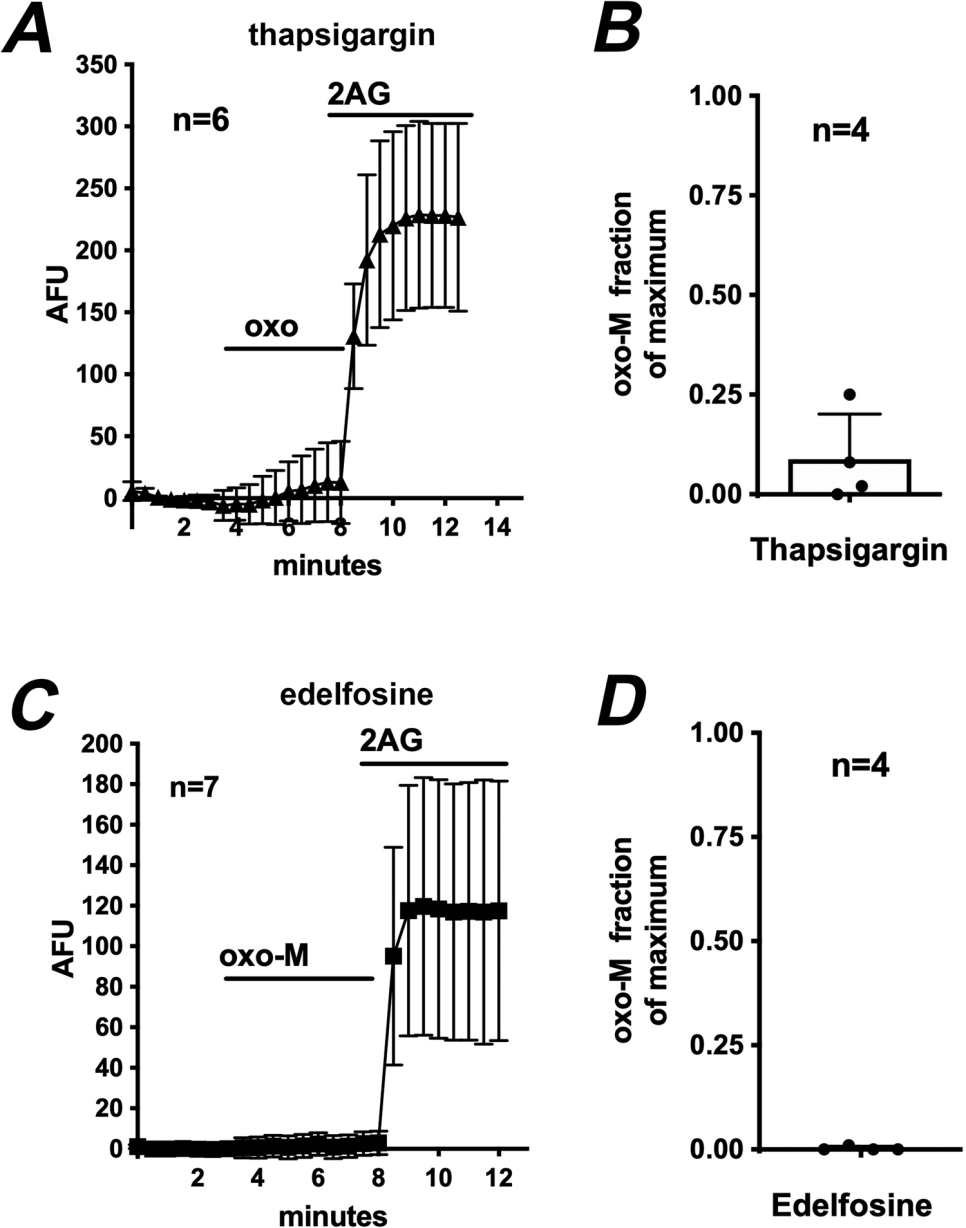
Replete internal calcium stores and PLC (phospholipase C) are required for M_3_‐mediated activation of the GRAB_eCB_ sensor *A* and *B*, overnight treatment with thapsigargin (20 nM) prevented the oxo‐M (oxotremorine‐M)‐stimulated response (0.087 ± 0.11, *n* = 4). *C* and *D* the PLC inhibitor edelfosine (20 µM for 5 min) also prevented the oxo‐M‐stimulated response (0.0025 ± 0.005, *n* = 4). *P* = 0.0449 and *P* = 0.0009, respectively, using unpaired *t* test *versus* oxo‐M responses in control cells. Each data point in B and D represents a pooled response from 5 to 9 cells. Data in figures are presented as values (±SD).

If the muscarinic stimulation of NAPE‐PLD activity occurs via G_q_‐coupled receptors, this implicates phospholipase C (PLC) and subsequent inositol triphosphate (IP_3_)‐mediated release of calcium from intracellular calcium stores. This sequence is shown schematically in Fig. 1. To test for a contribution from internal calcium stores, we tested the impact of thapsigargin (20 nM, overnight), a sarco/endoplasmic reticulum calcium ATPase (SERCA) pump blocker that depletes calcium stores. We found that treatment with thapsigargin substantially reduced the response to oxo‐M (Fig. 4*A,B*, oxo‐M response as a fraction of maximal activation (±SD): 0.087 ± 0.11, *n* = 4; unpaired *t* test *vs*. control oxo‐M responses, *P* = 0.0449, *t* = 2.53, df = 6). We tested for a PLC role by treating cells with edelfosine (20 µM) for 5 min. We found that this effectively ablated the subsequent oxo‐M response (Fig. 4*C,D*, oxo‐M response as a fraction of maximal activation (±SD): 0.0025 ± 0.005, *n* = 4; unpaired *t* test *vs*. control oxo‐M responses, *P* = 0.0009, *t* = 6.14, df = 6). Thus oxo‐M requires muscarinic receptors, full calcium stores and PLCβ to stimulate eCB production in HEK293 cells.

### Anandamide‐synthesizing and anandamide‐metabolizing proteins are expressed in murine lacrimal glands

**Figure 5:**
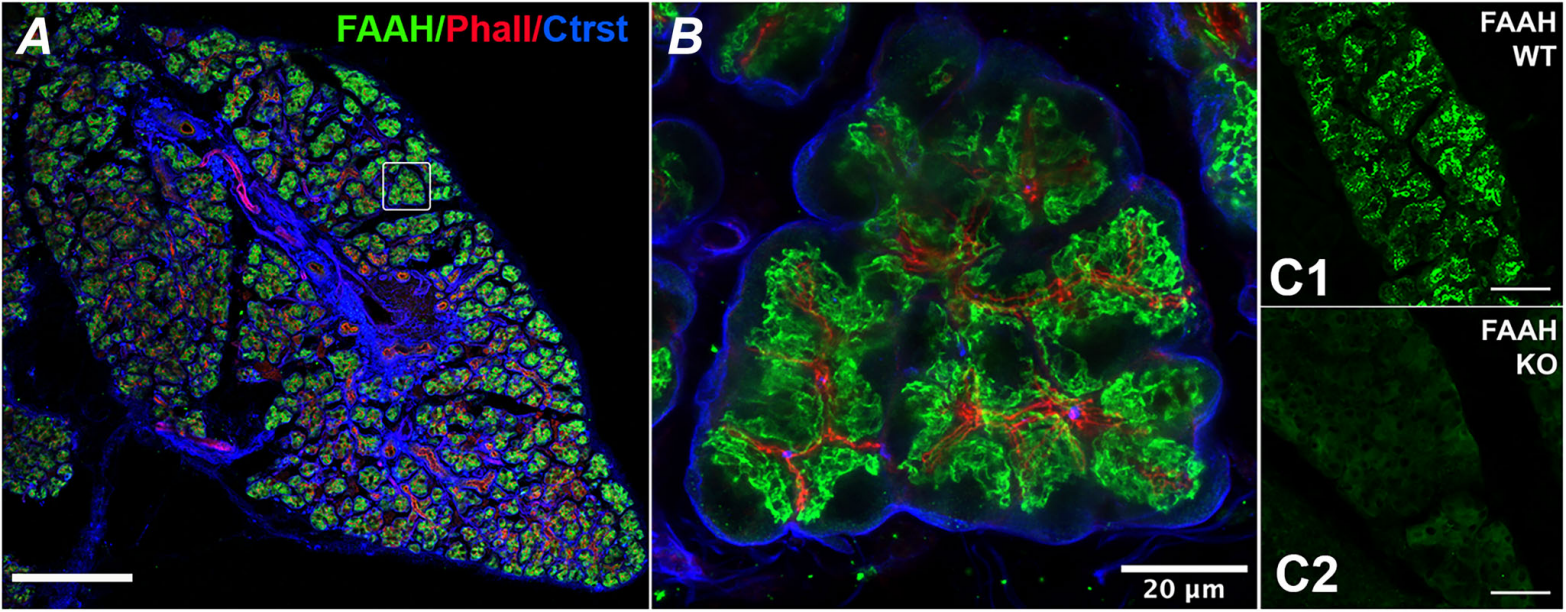
FAAH (fatty acid amide hydrolase) is expressed in acinar cells of the lacrimal gland *A*, lacrimal gland of female mouse stained for FAAH (green), phalloidin (red) and a counterstain (blue) shows that FAAH is expressed in all acini. *B*, higher magnification inset from panel A shows staining adjoins ducts within acini. *C*, FAAH staining is present in lacrimal gland from wild‐type (WT) but not FAAH knockout mice. Scale bar: (*A*) 200 µm; (*B*) 20 µm; (*C*) 100 µm.

As noted in the Introduction NAPE‐PLD is implicated in the synthesis of anandamide and related acylethanolamines. The enzymes FAAH (Cravatt et al., 1996) and *N*‐acylethanolamine‐hydrolysing acid amidase (NAAA) (Tsuboi et al., 2005) have each been demonstrated to metabolize anandamide. We have previously shown that FAAH protein is expressed in submandibular glands and that pharmacological inhibition of FAAH mimics CB1 activation to reduce salivation (Andreis et al., 2022). FAAH has also been reported to be expressed on the surface of calcium‐storing organelles such as smooth endoplasmic reticulum (Gulyas et al., 2004). Using immunohistochemistry we localized FAAH protein in lacrimal glands, finding that FAAH is abundantly expressed within acinar cells (Fig. 5*A,B*). FAAH staining was absent in FAAH knockout tissue (Fig. 5*C*).

**Figure 6:**
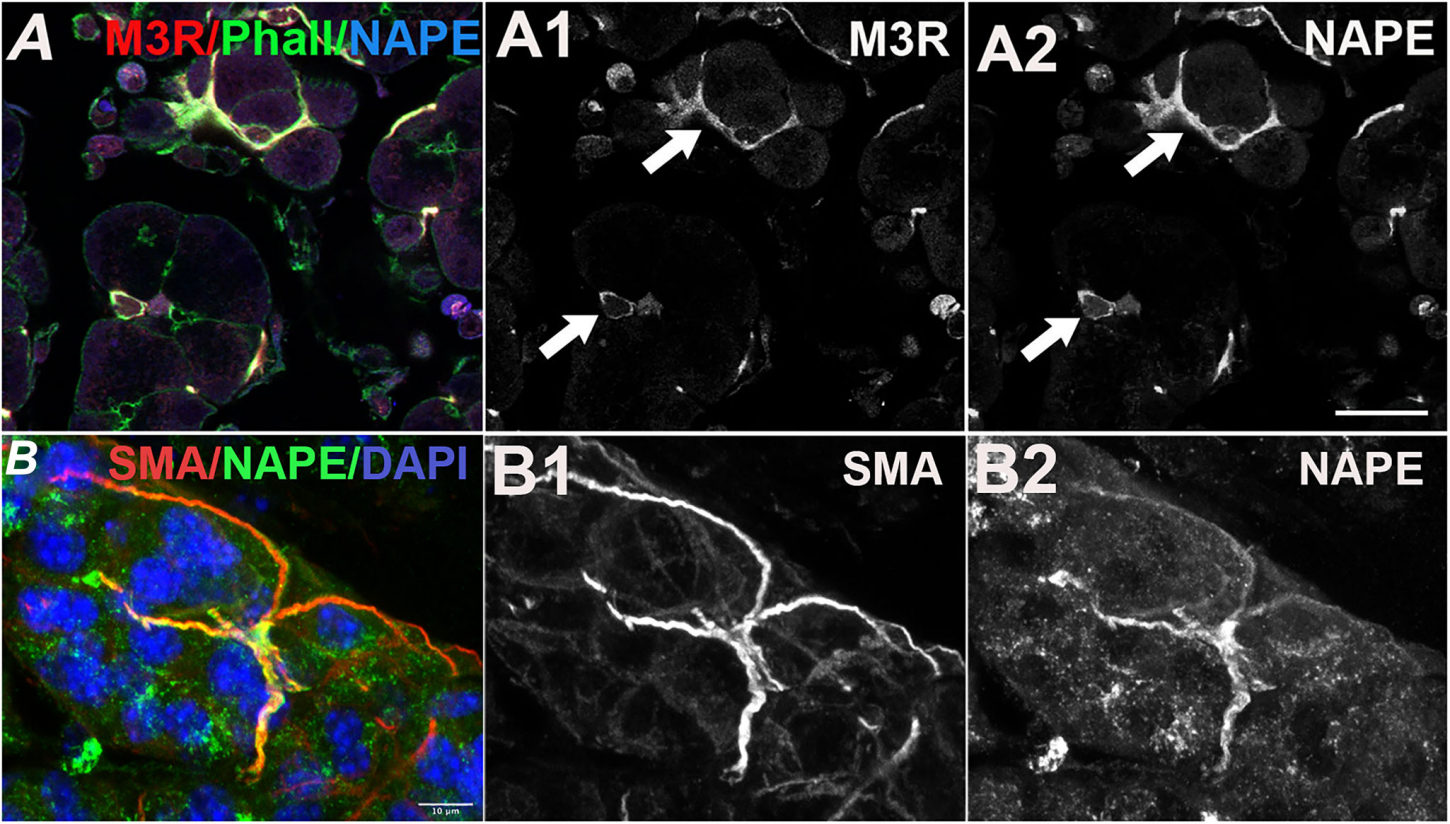
NAPE‐PLD (*N*‐arachidonoyl‐phosphatidylethanolamide phospholipase D) is co‐expressed with muscarinic M_3_ receptors in myoepithelial cells of the lacrimal gland *A*, immunohistochemistry staining of mouse lacrimal gland with NAPE‐PLD (blue), muscarinic M_3_ (red) and phalloidin (phalloidin) shows co‐expression of NAPE‐PLD and muscarinic M_3_ receptors. (A1) M_3_ staining from panel A. (A2) NAPE‐PLD staining from panel A. *B*, NAPE‐PLD (*N*‐arachidonoyl‐phosphatidylethanolamide phospholipase D, green) colocalizes with myoepithelial cell marker smooth muscle actin (SMA, red). Nuclei stained with DAPI (4',6‐diamidine‐2'‐phenylindole dihydrochloride). B1_SMA staining from panel B. (B2) NAPE staining from panel B. Scale bar: (*A*) 30 µm; (*B*) 10 µm.

We also evaluated the expression of NAPE‐PLD protein in lacrimal glands using an antibody previously validated in knockout tissue (Nyilas et al., 2008) as well as a muscarinic M_3_ receptor antibody that has been validated in knockout tissue. We found that NAPE‐PLD is colocalized with muscarinic M_3_ receptors (Fig. 6*A*) in what are likely myoepithelial cells. We additionally tested for co‐expression with the myoepithelial cell marker smooth muscle actin (SMA), though we also observed punctate SMA staining in some acinar cells using our SMA antibody (Fig. 6*B*).

**Figure 7:**
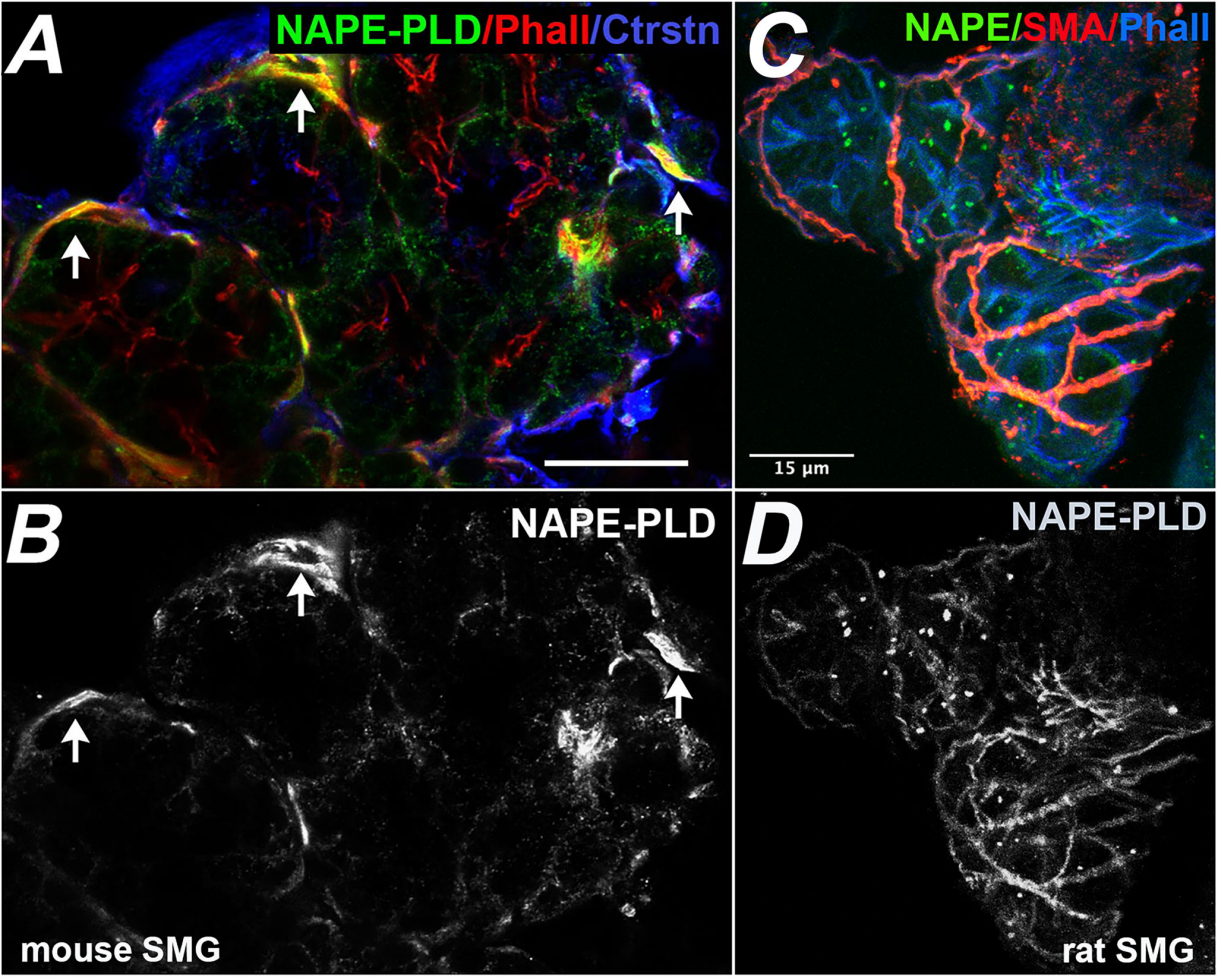
NAPE‐PLD (*N*‐arachidonoyl‐phosphatidylethanolamide phospholipase D) protein expression in myoepithelial cells of the submandibular gland of the mouse and rat *A*, immunohistochemistry staining in rat submandibular gland with NAPE‐PLD (green), phalloidin (red) and non‐specific counterstain (blue) shows expression of NAPE‐PLD in a myoepithelial cell‐like staining pattern. *B*, NAPE‐PLD staining from panel A. *C*, in rat submandibular gland NAPE‐PLD (green) overlaps substantially with myoepithelial cell marker smooth muscle actin (SMA, red). *D*, NAPE‐PLD staining from panel C. Scale bar: (*A* and *B*) 10 µm; (*C* and *D*) 15 µm.

In our study of cannabinoid regulation of the function of SMGs, we reported FAAH protein expression similar to what we report here. We have used the same NAPE‐PLD antibody to test for protein expression in mouse submandibular glands. We included rat tissue because our SMA antibody, generated against a mouse epitope, sometimes yields an artifactual stain in mouse but produces artifact‐free staining in rat tissue. The NAPE‐PLD staining labels myoepithelial cells similarly in both mouse (Fig. 7*A,B*) and rat (Fig. 7*C,D*).

### Muscarinic activation of acutely dissected lacrimal gland or SMG elicits an eCB sensor response

**Figure 8:**
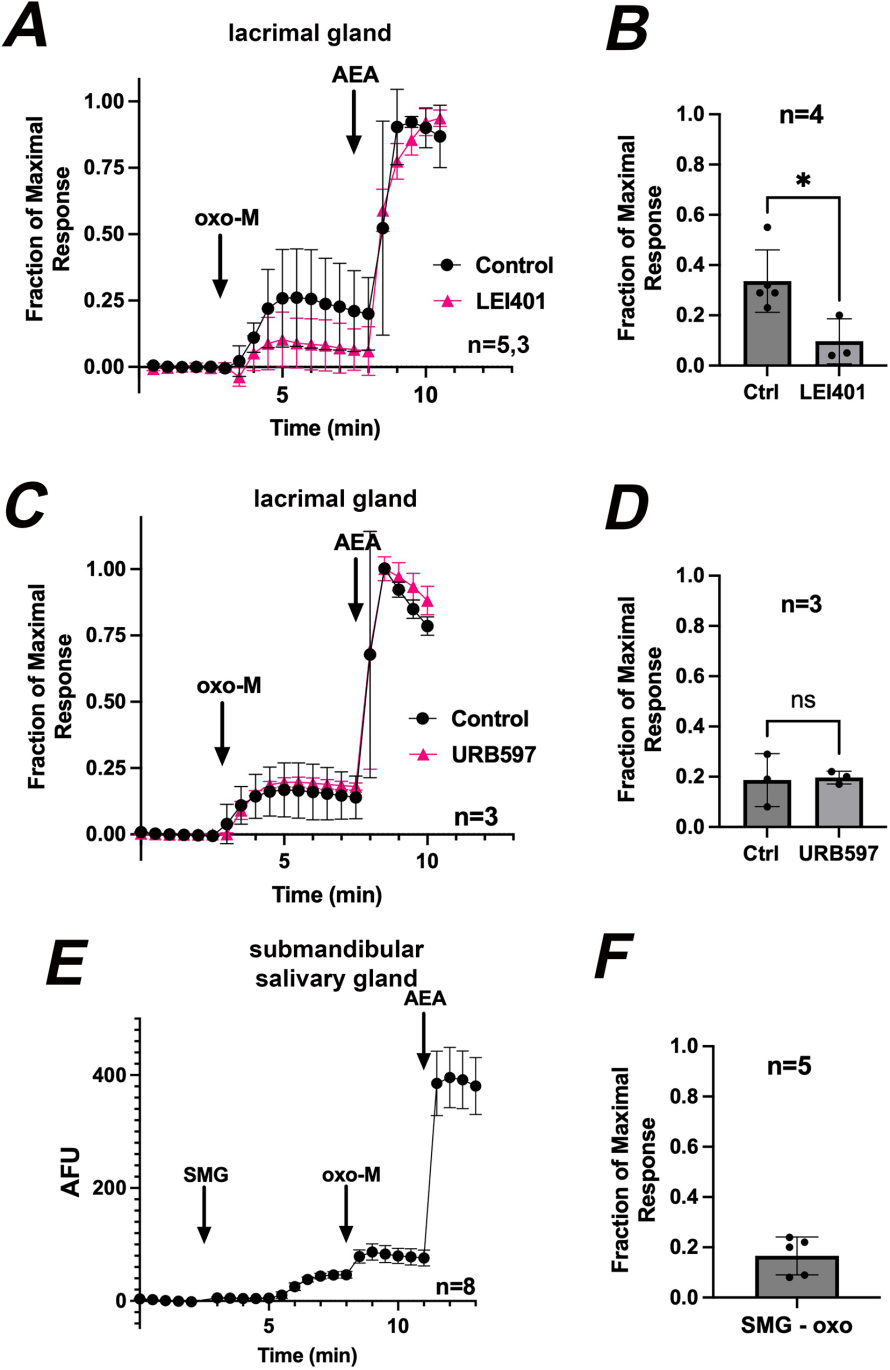
Muscarinic stimulation of lacrimal and salivary gland elicits an endocannabinoid (eCB) sensor response *A*, time course of fluorescence in HEK‐GRAB_eCB3.0_ responses to lacrimal gland activation by muscarinic agonist oxo‐M (oxotremorine‐M, 10 µM) followed by anandamide (30 µM). Values are normalized to maximal response to AEA (arachidonoylethanolamide) treatment at the end of experiment. In cells treated with NAPE‐PLD (*N*‐arachidonoyl‐phosphatidylethanolamide phospholipase D) blocker LEI401 (3 µM), responses to oxo‐M are strongly diminished. *B*, bar graph compares maximal responses to oxo‐M (as a fraction of maximal AEA response) in control *versus* LEI401‐treated cells. *C*, time courses for lacrimal gland sections treated with oxo‐M and AEA as earlier but testing for the effect of FAAH (fatty acid amide hydrolase) blocker URB597 (300 nM). *D*, bar graph shows the effect of FAAH blocker URB597 as earlier. *E*, sample time course showing eCB3.0 sensor response to introduction of submandibular gland explant followed by stimulation with oxo‐M and general stimulation with AEA (30 µM). *F*, bar graph summarizing submandibular gland responses to oxo‐M. *P = 0.03, unpaired *t* test. Each data point in panels B, D and F represents a pooled response from 5 to 9 cells. Data are shown as values (±SD).

We next tested whether acutely dissected sections of lacrimal glands could be stimulated *ex vivo* to release eCBs. HEK293‐GRAB_eCB_‐expressing cells were grown as mentioned earlier in individual wells of a 48‐well plate. Lacrimal glands were obtained from adult female mice, with the capsules dissected away, and then cut into three sections. Each gland section was added to the bottom of a well shortly before testing. Gland sections generally remained in place during the experiment. In a few instances where the sections moved, the experiment was repeated with a fresh gland section. After a baseline time course was obtained, media containing oxo‐M (final concentration 10 µM) was added to the well. The GRAB_eCB_ sensor response was observed for a further 5 min followed by the addition of a high concentration (30 µM) of anandamide (AEA) to elicit a maximal response. We found that oxo‐M produced a reliable response that peaked at ∼2 min after treatment (Fig. 8*A,B*). This effect of oxo‐M decreased if glands were pre‐treated with NAPE‐PLD blocker LEI401 (3 µM, 30 min; Fig. 8*A,B*; *P* = 0.0280 (*t* = 2.8, df = 6) using unpaired *t* test *vs*. oxo‐M controls).

In separate experiments we tested whether treatment with the FAAH blocker URB597 would alter the amplitude or time course of lacrimal gland oxo‐M responses. The rationale was that the abundant FAAH in acinar cells may serve as a sump by rapidly metabolizing anandamide. Preventing this metabolism might significantly enhance the oxo‐M‐elicited response. However we did not see any effect of treatment with FAAH blocker URB597 (300 nM, 30 min) relative to same‐day controls (Fig. 8*C*,*D*; *P* = 0.880 (*t* = 0.16, df = 4) using unpaired *t* test).

We have previously shown that submandibular glands also express a CB1‐based cannabinoid signalling system whose activation inhibits salivation (Andreis et al., 2022). Submandibular glands also express FAAH, in a similar expression pattern to that observed in lacrimal glands (Andreis et al., 2022) (i.e. within acinar cells) and NAPE‐PLD (Fig. 7). We therefore tested whether submandibular gland explants (from either sex) could be similarly stimulated by a muscarinic agonist to activate the GRAB_eCB_ sensor, finding that oxo‐M treatment also elicited an eCB sensor response (Fig. 8*E,F*; *P* = 0.008 (*t* = 4.9, df = 4), 1 sample *t* test *vs*. baseline).

We additionally tested whether the acylethanolamines OEA, PEA or LEA would directly activate the HEK293‐GRAB_eCB_ sensor. We found that OEA, PEA and LEA each did not activate the HEK293‐GRAB_eCB3.0_ sensor at 10 µM (data not shown, OEA (fraction of maximum ± SD): 0.0133 ± 0.0057, *n* = 3; PEA: 0.0466 ± 0.025, *n* = 3; LEA: 0.0433 ± 0.127, *n* = 3).

### eCB‐specific sensors suggest muscarinic stimulation of lacrimal glands produces acylethanolamides

**Figure 9:**
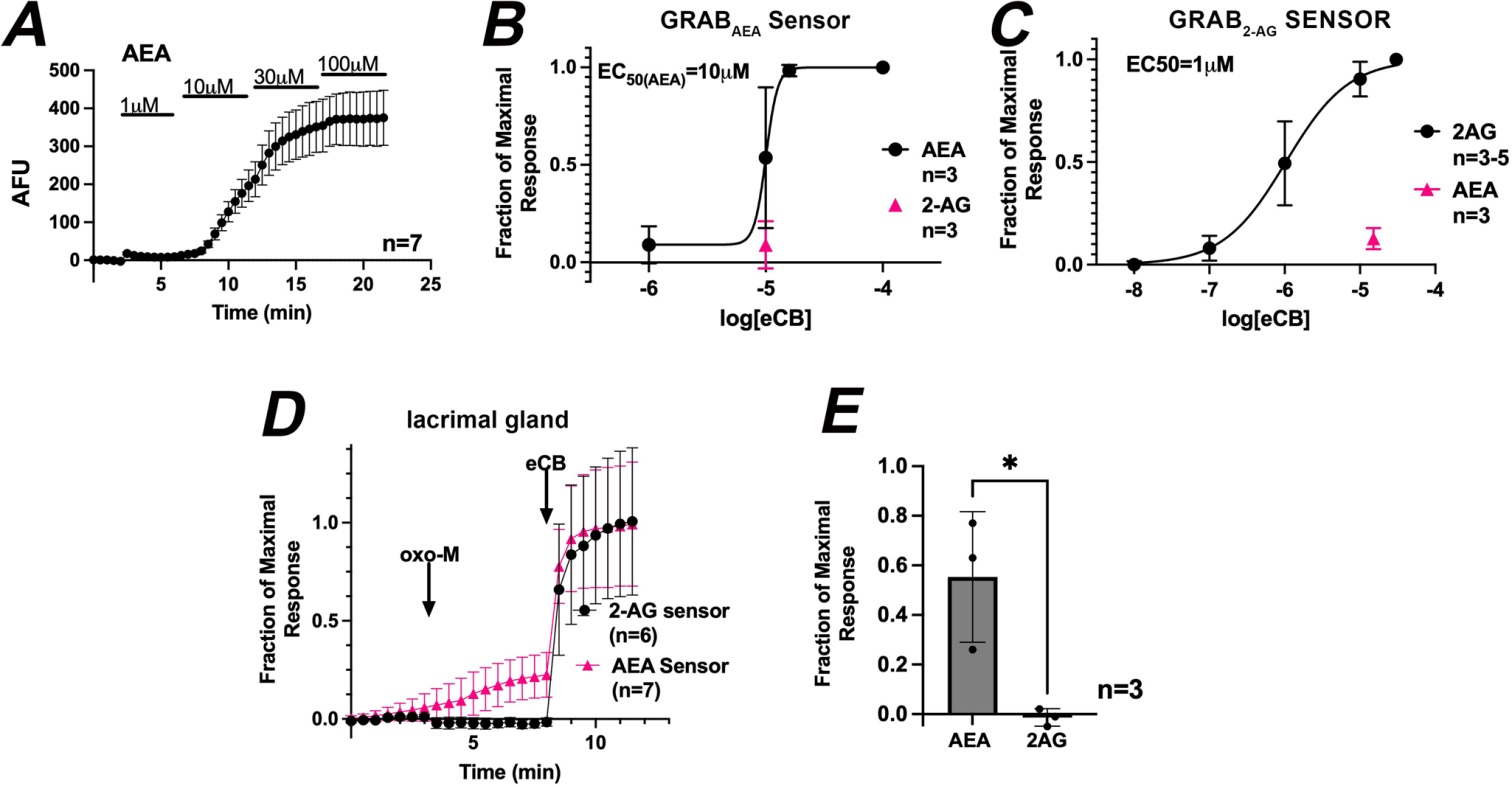
Muscarinic stimulation of lacrimal gland elicited a response in AEA (arachidonoylethanolamide)‐ but not 2‐AG (2‐arachidonoylglycerol)‐selective eCB (endocannabinoid) sensors *A*, sample time course showing the effects of increasing concentrations of AEA on GRAB_AEA_ responses in HEK293 cells. *B*, dose response for AEA and response to 10 µM 2‐AG at the GRAB_AEA_ sensor. *C*, summary dose response for 2‐AG and response to 15 µM AEA at the GRAB_2AG_ sensor. *D*, sample time course showing AEA‐ and 2‐AG‐specific sensor responses to lacrimal gland explant activated by oxo‐M (oxotremorine‐M) followed by direct activation with AEA (30 µM) or 2‐AG (10 µM). *E*, bar graph summarizes lacrimal gland‐induced responses to the 2 different eCB‐specific sensors, *n* = 3. ^*^ *P* = 0.02 using unpaired *t* test. Each data point in panel E represents a pooled response from 5 to 9 cells. Data are shown as values (±SD).

To examine the production of endocannabinoids (eCBs) by lacrimal glands with greater resolution, we used GRAB_eCB_ sensors designed to be selective for anandamide (GRAB_AEA_) or 2‐AG (GRAB_2‐AG_). We first confirmed the selectivity of these sensors in HEK293‐transfected cells. Figure 9 shows that anandamide stimulated responses in the HEK293‐GRAB_AEA_ cells with an EC_50_ value of 10 µM, whereas 10 µm of 2‐AG was not effective (Fig. 9*A,B*; EC_50_ for AEA (95% confidence interval (CI)): 10.0 µM (2.6–13.1 µM); 2‐AG as a fraction of maximal AEA response (±SD): 0.090 ± 0.121, *n* = 3). It should be noted that we recorded values at 5 min postapplication. Figure 9*A* shows that the responses to some concentrations had not yet reached a plateau by 5 min. The EC_50_ value for AEA is therefore likely an underestimate of its potency at this sensor. Turning to the GRAB_2AG_ sensor we found that 2‐AG was more potent at the GRAB_2AG_ sensor with an EC_50_ value of 1 µM; 15 µM AEA induced only a modest response (Fig. 9*C*; EC_50_ for 2‐AG (95% CI): 1.04 µM (0.70–1.51 µM); AEA as a fraction of maximal 2‐AG response (±SD): 0.126 ± 0.0513, *n* = 3).

We repeated the aforementioned experiments, introducing acutely dissected lacrimal glands to HEK293 cells that expressed either the GRAB_AEA_ or GRAB_2AG_ sensor. The experimental set‐up differed from that of previous experiments in that GRAB_eCB_‐expressing cells were grown on poly‐d‐lysine‐coated coverslips and transferred to glass‐bottom Petri dishes for experiments. The cover glass diameter of 25 mm meant that the well was larger than a given well of a 48‐well plate. In initial experiments we found that stimulated glands did not reliably respond to oxo‐M. Considering the different experimental parameters and the previously reported benefits of including BSA as a carrier for eCBs (Singh et al., 2023), we found that the inclusion of 0.2% BSA for lacrimal glands treated with oxo‐M now reliably elicited a response in HEK293 cells transfected with the GRAB_AEA_ sensor. When the experiment was repeated with HEK293 cells expressing the GRAB_2AG_ sensor, no oxo‐M response was observed despite the robust response elicited by 2‐AG (10 µM) at the end of the experiment (Fig. 9*D,E*; *P* = 0.021, *t* = 3.69, df = 4, unpaired *t* test).

### GPCR stimulation of NAPE‐PLD is modular

**Figure 10:**
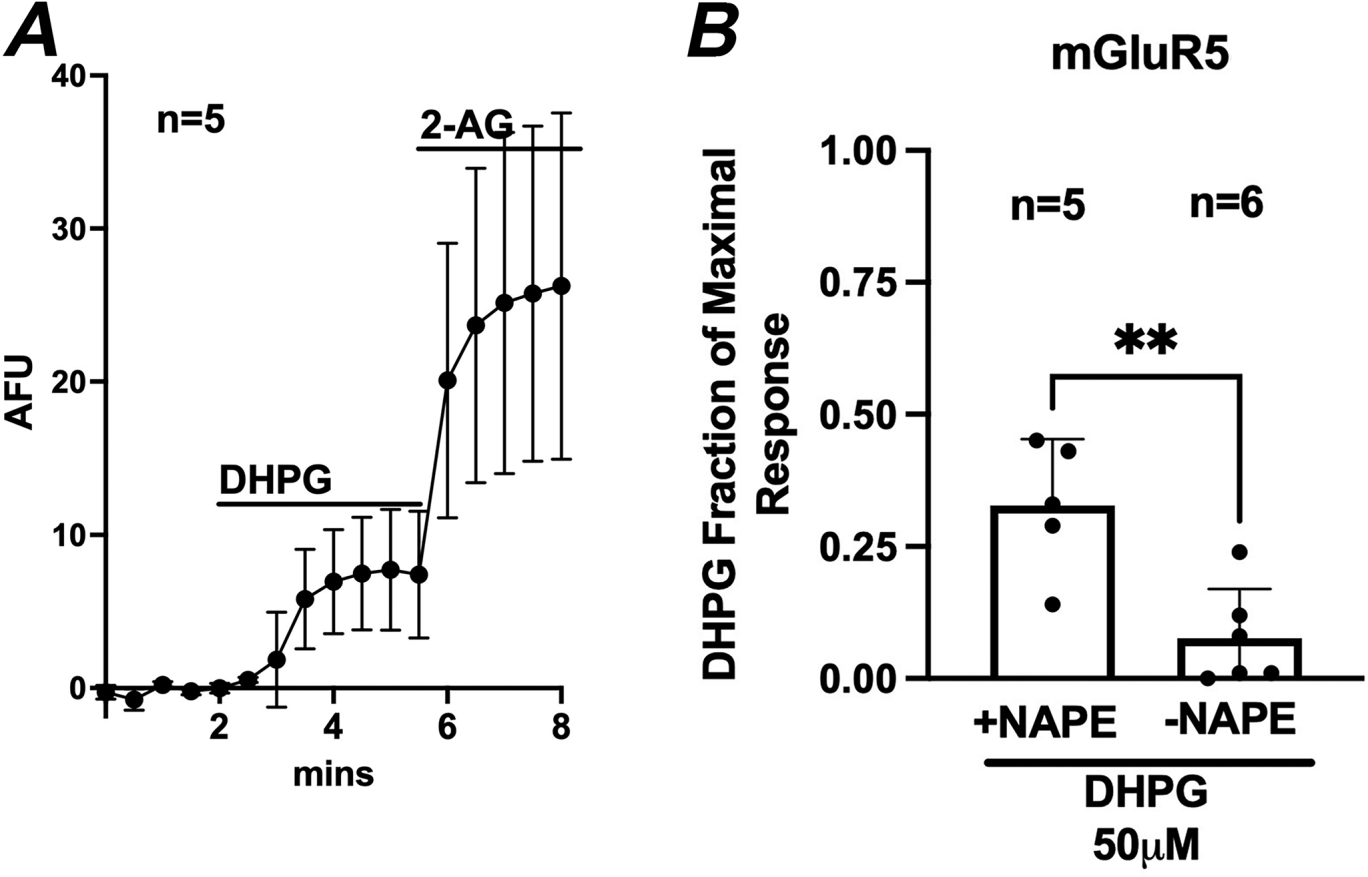
GPCR stimulation of NAPE‐PLD (*N*‐arachidonoyl‐phosphatidylethanolamide phospholipase D) is modular *A*, sample time course shows that DHPG (3,5‐dihydrophenylglycine, 50 µM) activation in HEK293 cells co‐expressing mGluR5 receptors, NAPE‐PLD and GRAB_eCB3.0_ stimulates an endocannabinoid (eCB) sensor response. *B*, averaged responses to DHPG as a fraction of subsequent maximal activation in HEK293 cells with and without NAPE‐PLD (+NAPE/–NAPE), *n* = 5, 6. ^**^ *P* = 0.0026 using unpaired *t* test. Data are shown as values (±SD).

We have thus far focused on muscarinic M_3_ receptors to stimulate anandamide synthesis. One feature of synthesis of the other eCB 2‐AG by DAGLα is its modularity. In principle any G_q_‐coupled receptor could stimulate 2‐AG production as long as the synthetic enzymes were present. Several GPCRs are implicated in 2‐AG production, including group I metabotropic glutamate receptors (mGluR1 and mGluR5). To test whether NAPE‐PLD activation may offer a similar modularity, we transfected HEK293 cells with mGluR5, NAPE‐PLD and GRAB_eCB_. We found that treatment of these cells with the mGluR5 receptor agonist 3,5‐dihydrophenylglycine (DHPG, 50 µM) now elicited an eCB sensor response similar to that observed in response to oxo‐M (Fig. 10*A,B*; DHPG (50 µM) response in NAPE+ cells (fraction of maximum ± SD): 0.328 ± 0.124, *n* = 5; in NAPE– cells: 0.0767 ± 0.0930, *n* = 6). This response was absent in cells expressing only mGluR5 and GRAB_eCB_ (Fig. 10*B*; DHPG responses in NAPE+ *vs*. NAPE– cells using unpaired *t* test: *P* = 0.0026; *t* = 4.36 df = 8).

## Discussion

The cannabinoid signalling system regulates many important physiological functions, including tearing and salivation (Andreis et al., 2022; Murataeva, Mattox et al., 2024; Thayer et al., 2020). One curiosity of the cannabinoid signalling system is that it makes use of two messengers, anandamide and 2‐AG, that are synthesized and metabolized by distinct sets of enzymes. The synthetic pathway(s) and physiological roles for anandamide are still incompletely understood, but anandamide is implicated in salivation (Andreis et al., 2022) and tearing. We employed immunohistochemistry and GRAB_eCB_ sensors to investigate anandamide production in cell lines and lacrimal/salivary glands and now report several novel findings. First an endogenously expressed G_q_‐coupled muscarinic receptor in HEK293 cells can stimulate the formation of anandamide via NAPE‐PLD, utilizing a pathway requiring both PLC and replete internal calcium stores. This validates a previously proposed mechanism to initiate the production of anandamide (van der Stelt et al., 2005), one that may be especially relevant to non‐neuronal cells. We further demonstrate that this muscarinic stimulation of eCB production occurs natively in lacrimal and salivary glands. This likely involves the production of anandamide and perhaps other acylethanolamines by NAPE‐PLD. Finally we demonstrate that a second G_q_‐coupled receptor mGluR5 similarly activates NAPE‐PLD in HEK293 cells, suggesting that this form of signalling is modular.

As noted the canonical eCB signalling system makes use of two lipid messengers. Though anandamide was identified first it was 2‐AG that saw more rapid strides in the understanding how its synthesis was regulated. A flurry of publications showed that 2‐AG could be synthesized ‘on demand’ either by neuronal depolarization or by activation of G_q_‐coupled GPCRs (reviewed in Murataeva et al., 2014). Several forms of, chiefly retrograde, neuronal plasticity were found to employ 2‐AG (reviewed in Kano et al., 2009). Meanwhile the regulatory details of AEA synthesis remained a mystery. NAPE‐PLD was an early candidate for the release of anandamide from a phospholipid precursor *N*‐arachidonoyl‐phosphatidylethanolamine (Okamoto et al., 2004; Sugiura et al., 1996), shown schematically in Fig. 1. However results from the first‐reported NAPE‐PLD knockout mice argued against a role for this enzyme because anandamide levels were slightly altered in the brains of these mice (Leung et al., 2006). What followed was a prolonged effort to identify alternative means of producing anandamide (e.g. Simon & Cravatt, 2006). Another 10 years would pass before results from a different strain of NAPE‐PLD knockout mice confirmed a central role for this enzyme (Leishman et al., 2016). The centrality of NAPE‐PLD in anandamide biosynthesis was thus established nearly 25 years after the initial identification of anandamide as an eCB. In the interim the absence of a clear target hindered the development of relevant pharmacological tools; it was only in 2020 that the first potent NAPE‐PLD inhibitor was described (Mock et al., 2020), nearly 15 years after DAGL blockers for 2‐AG became commonplace (Bisogno et al., 2006).

Before proceeding it must be emphasized that the NAPE‐PLD enzyme synthesizes acylethanolamines, a family of lipids that includes anandamide but also other bioactive lipids such as OEA (Ohno‐Shosaku et al., 2002) and PEA. Although anandamide has received the lion's share of attention as one of two eCBs, the question of whether other acylethanolamines alone, or in combination (i.e. as an entourage), activate cannabinoid receptors is not fully resolved. We found that OEA, PEA and LEA were each inactive at the GRAB_eCB_ sensor at 10 µM, indicating that these lipids are unlikely to account for the GRAB_eCB_ sensor responses to oxo‐M administration in lacrimal glands. However because we did not exhaustively test other acylethanolamines alone or as a group, it is possible that the responses in either the HEK293 cell model or exocrine glands are due to some combination of acylethanolamines. A second consideration concerns the endogenous expression of NAPE‐PLD in HEK293 cells. The report on endogenous activity of NAPE‐PLD in HEK293 by Guo et al. (2013) raises the question to what extent endogenous NAPE‐PLD contributes to the responses observed here. As noted in results we tested oxo‐M in the absence of transfected enzymes (but with the GRAB_eCB3.0_ sensor) and observed no responses. The endogenous NAPE‐PLD activity in the Guo study was observed over the course of >6 h and so may have been relatively low and insufficient to observe the acute stimulated responses that are detectable by the GRAB sensor.

NAPE‐PLD has a binding site for bile acids (Magotti et al., 2015). Different bile acids may substantially increase or decrease the activity of the NAPE‐PLD enzyme (Margheritis et al., 2016). NAPE‐PLD is described as a zinc metallohydrolase and has a zinc‐lined catalytic region that is likely necessary for the enzymatic activity of NAPE‐PLD (Magotti et al., 2015; Wang et al., 2006). Although zinc is a co‐factor it is unclear whether zinc levels regulate the activity of NAPE‐PLD; Ueda et al. have reported that the addition of Zn^2+^ reduced enzyme activity (Ueda et al., 2005). Early work demonstrated a requirement for calcium for anandamide production (Di Marzo et al., 1994), thereby raising two questions: (1) what is the target of the increased intracellular calcium, and (2) what is the source of calcium? Early studies demonstrated that increasing calcium stimulated the activity of the enzyme NAT, which produces the precursor, NAPE, for NAPE‐PLD to act on (Natarajan et al., 1983; Schmid et al., 1983). Subsequent studies using purified NAPE‐PLD suggested that NAPE‐PLD itself can be activated by calcium (Ueda et al., 2001; Wang et al., 2006), though the conclusion drawn was that NAPE‐PLD is constitutively active and that NAT remains the rate‐limiting step (reviewed in Okamoto et al., 2007). Thus this places NAPE‐PLD in the role of a passive participant of the synthetic process. If the upstream NAT is the rate‐limiting step, interventions might usefully block NAPE‐PLD to decrease anandamide production, but NAPE‐PLD activation would be ineffectual to increase anandamide production because NAT activity limits the production of NAPE and hence anandamide. This arrangement differs qualitatively from the synthesis of 2‐AG where the synthetic enzyme DAGLα can be directly regulated (reviewed in Kano et al., 2009; Shonesy et al., 2020).

What then is the source of calcium? In neurons and other excitable cells, a likely mechanism involves neuron depolarization‐induced influx through voltage‐sensitive calcium channels. What about non‐excitable cells? A study in HEK293 cells showed that the depletion of calcium from intracellular stores diminished anandamide production (Ligresti et al., 2004). A subsequent review referred to this and unpublished data pointing to a role for PLC (Ligresti et al., 2004; van der Stelt et al., 2005) and explicitly proposed that G_q_‐coupled muscarinic or metabotropic receptors might stimulate anandamide synthesis. Our work appears to bear out this proposed arrangement and highlights calcium release from internal stores as a suitable source of calcium for anandamide production.

Our finding that two exocrine glands employ muscarinic activation to stimulate NAPE‐PLD‐dependent production of acylethanolamines demonstrates the physiological relevance of this means of producing acylethanolamines such as anandamide, especially in non‐neuronal cells. In submandibular glands we observed abundant FAAH expression in acinar cells and proposed that locally produced anandamide could act on CB1 receptors on parasympathetic inputs to inhibit ACh release, thereby reducing salivation (Andreis et al., 2022). The action of tetrahydrocannabinol (THC) on these CB1 receptors may account for the dry mouth often reported by cannabis users. The role of endogenous anandamide would represent a form of feedback inhibition not unlike what has been observed for 2‐AG in the CNS (Kano et al., 2009), albeit with anandamide as the messenger. Our results suggest that anandamide is the likely product of local muscarinic activation in lacrimal and perhaps salivary glands though again our results do not rule out a contribution from other acylethanolamines. Of the five muscarinic receptors three (M1, M_3_ and M5) are G_q_ coupled (Haga, 2013). Previous studies of muscarinic regulation of tearing and salivation favour roles for muscarinic M_3_ receptors (e.g. Lemullois et al., 1996). An M_3_ role may extend to other exocrine glands (e.g. Schiavone & Brambilla, 1991, for sweating). Our finding that lacrimal glands also possess the requisite machinery to produce eCBs via this mechanism underscores the physiological relevance of this means of producing anandamide. We have recently shown that a cannabinoid system including NAPE‐PLD is active in murine sweat glands (Murataeva et al., 2026), so it is possible that a similar arrangement holds in sweat glands. A recently published paper also links NAPE‐PLD to hypertension (Chiarugi et al., 2025), proposing that NAPE‐PLD is a target for thiazide‐class antihypertensives. Metabotropic regulation of calcium microdomains may impact NAPE‐PLD function. In principle the reported bile acid interference with cholinergic airway constriction might be due to the regulation of muscarinic activation of NAPE‐PLD activity (Urso et al., 2020). Though speculative these examples highlight some of the diverse potential physiological implications of this signalling pathway.

One interesting and attractive feature of GPCR‐induced production of anandamide is that it is modular. In principle any G_q_‐coupled GPCR that is sufficiently close to the enzyme could, when activated, elicit NAPE‐PLD synthesis of acylethanolamines. This has been shown to be the case for 2‐AG synthesis, which has been shown to be stimulated by muscarinic, metabotropic glutamate, serotonin and orexin receptors (reviewed in Jung et al., 2012; Murataeva et al., 2014). We found that metabotropic glutamate receptors can also couple to NAPE‐PLD‐mediated anandamide synthesis and contend that it is therefore possible, even likely, that other G_q_‐coupled receptors may plug into this synthetic pathway.

### Conclusion

In summary these experiments demonstrate that anandamide synthesis can be stimulated by the activation of a G_q_‐coupled GPCR both in intact cells and in exocrine explants. This means of stimulating AEA production may be especially relevant in non‐neuronal cells and tissues using calcium released from internal stores. We find that this means of producing anandamide is employed in lacrimal glands and likely SMGs. This mechanism of stimulating anandamide production by G_q_‐coupled GPCRs may be widely employed throughout the body.

## Additional information

## Competing interests

The authors declare that they have no competing interests.

## Author contributions

Conceptualization: N.M. and A.S.; formal analysis: A.S. and N.M.; investigation: C.S., E.H., A.S., i.m., N.M.; writing – original draft preparation: A.S.; writing – review and editing: N.M., K.M.; resources: Y.L., R.C., S.C., J.W.‐M.; supervision: A.S. and N.M.; project administration:, A.S.; All authors have read and agreed to the published version of the manuscript.

## Funding

Supported in part by the National Institutes of Health grant AT011162 (K.M.).

## Generative AI Statement

No AI tools were used in the preparation of this manuscript.

## Supporting information


Peer Review History

## Data Availability

Data used in the manuscript may be found within the manuscript.

## References

[tjp70812-bib-0001] Andreis, K. , Billingsley, J. , Naimi Shirazi, K. , Wager‐Miller, J. , Johnson, C. , Bradshaw, H. , & Straiker, A. (2022). Cannabinoid CB1 receptors regulate salivation. Scientific Reports, 12(1), 14182. 35986066 10.1038/s41598-022-17987-2 PMC9391487

[tjp70812-bib-0002] Atwood, B. K. , Lopez, J. , Wager‐Miller, J. , Mackie, K. , & Straiker, A. (2011). Expression of G protein‐coupled receptors and related proteins in HEK293, AtT20, BV2, and N18 cell lines as revealed by microarray analysis. BioMed Central Genomics, 12(1), 14. 21214938 10.1186/1471-2164-12-14 PMC3024950

[tjp70812-bib-0003] Bisogno, T. , Cascio, M. G. , Saha, B. , Mahadevan, A. , Urbani, P. , Minassi, A. , Appendino, G. , Saturnino, C. , Martin, B. , & Razdan, R. (2006). Development of the first potent and specific inhibitors of endocannabinoid biosynthesis. Biochimica Et Biophysica Acta, 1761(2), 205–212. 16466961 10.1016/j.bbalip.2005.12.009

[tjp70812-bib-0004] Bisogno, T. , Howell, F. , Williams, G. , Minassi, A. , Cascio, M. G. , Ligresti, A. , Matias, I. , Schiano‐Moriello, A. , Paul, P. , Williams, E. J. , Gangadharan, U. , Hobbs, C. , Di Marzo, V. , & Doherty, P. (2003). Cloning of the first sn1‐DAG lipases points to the spatial and temporal regulation of endocannabinoid signaling in the brain. Journal of Cell Biology, 163(3), 463–468. 14610053 10.1083/jcb.200305129 PMC2173631

[tjp70812-bib-0005] Brandt, J. E. , Priori, R. , Valesini, G. , & Fairweather, D. (2015). Sex differences in Sjogren's syndrome: A comprehensive review of immune mechanisms. Biology of Sex Differences, 6(1), 19. 26535108 10.1186/s13293-015-0037-7 PMC4630965

[tjp70812-bib-0006] Cai, R. , Cai, S. , Dong, A. , & Li, Y. (2024). Monitoring anandamide (AEA) and 2‐arachidonoylglycerol (2‐AG) dynamics with selective endocannabinoid GRAB sensors. Society for Neuroscience.

[tjp70812-bib-0007] Chiarugi, S. , Margheriti, F. , De Lorenzi, V. , Martino, E. , Margheritis, E. G. , Moscardini, A. , Marotta, R. , Chaves‐Sanjuan, A. , Del Seppia, C. , Federighi, G. , Lapi, D. , Bandiera, T. , Rapposelli, S. , Scuri, R. , Bolognesi, M. , & Garau, G. (2025). NAPE‐PLD is target of thiazide diuretics. Cell Chemical Biology, 32(3), 449–462. 39999832 10.1016/j.chembiol.2025.01.008

[tjp70812-bib-0008] Cravatt, B. F. , Demarest, K. , Patricelli, M. P. , Bracey, M. H. , Giang, D. K. , Martin, B. R. , & Lichtman, A. H. (2001). Supersensitivity to anandamide and enhanced endogenous cannabinoid signaling in mice lacking fatty acid amide hydrolase. Proceedings of the National Academy of Sciences of the United States of America, 98(16), 9371–9376. 11470906 10.1073/pnas.161191698 PMC55427

[tjp70812-bib-0009] Cravatt, B. F. , Giang, D. K. , Mayfield, S. P. , Boger, D. L. , Lerner, R. A. , & Gilula, N. B. (1996). Molecular characterization of an enzyme that degrades neuromodulatory fatty‐acid amides. Nature, 384(6604), 83–87. 8900284 10.1038/384083a0

[tjp70812-bib-0010] Daiyasu, H. , Osaka, K. , Ishino, Y. , & Toh, H. (2001). Expansion of the zinc metallo‐hydrolase family of the beta‐lactamase fold. Federation of European Biochemical Societies Letters, 503(1), 1–6. 11513844 10.1016/s0014-5793(01)02686-2

[tjp70812-bib-0011] Devane, W. A. , Hanuš, L. , Breuer, A. , Pertwee, R. G. , Stevenson, L. A. , Griffin, G. , Gibson, D. , Mandelbaum, A. , Etinger, A. , & Mechoulam, R. (1992). Isolation and structure of a brain constituent that binds to the cannabinoid receptor. Science, 258(5090), 1946–1949. 1470919 10.1126/science.1470919

[tjp70812-bib-0012] Di Marzo, V. , Fontana, A. , Cadas, H. , Schinelli, S. , Cimino, G. , Schwartz, J. C. , & Piomelli, D. (1994). Formation and inactivation of endogenous cannabinoid anandamide in central neurons. Nature, 372(6507), 686–691. 7990962 10.1038/372686a0

[tjp70812-bib-0013] Dong, A. , He, K. , Dudok, B. , Farrell, J. S. , Guan, W. , Liput, D. J. , Puhl, H. L. , Cai, R. , Wang, H. , Duan, J. , Albarran, E. , Ding, J. , Lovinger, D. M. , Li, B. , Soltesz, I. , & Li, Y. (2022). A fluorescent sensor for spatiotemporally resolved imaging of endocannabinoid dynamics in vivo. Nature Biotechnology, 40(5), 787–798. 10.1038/s41587-021-01074-4 PMC9091059 34764491

[tjp70812-bib-0014] Dvorakova, M. , Bosquez‐Berger, T. , Billingsley, J. , Murataeva, N. , Woodward, T. , Leishman, E. , Zimmowitch, A. , Gibson, A. , Wager‐Miller, J. , Cai, R. , Cai, S. , Ware, T. , Hsu, K. L. , Li, Y. , Bradshaw, H. , Mackie, K. , & Straiker, A. (2025). Acetaminophen inhibits diacylglycerol lipase synthesis of 2‐arachidonoyl glycerol: Implications for nociception. Cell Reports Medicine, 6(6), 102139. 40381619 10.1016/j.xcrm.2025.102139 PMC12208326

[tjp70812-bib-0015] Gulyas, A. I. , Cravatt, B. F. , Bracey, M. H. , Dinh, T. P. , Piomelli, D. , Boscia, F. , & Freund, T. F. (2004). Segregation of two endocannabinoid‐hydrolyzing enzymes into pre‐ and postsynaptic compartments in the rat hippocampus, cerebellum and amygdala. European Journal of Neuroscience, 20(2), 441–458. 15233753 10.1111/j.1460-9568.2004.03428.x

[tjp70812-bib-0016] Guo, L. , Gragg, S. D. , Chen, Z. , Zhang, Y. , Amarnath, V. , & Davies, S. S. (2013). Isolevuglandin‐modified phosphatidylethanolamine is metabolized by NAPE‐hydrolyzing phospholipase D. Journal of Lipid Research, 54(11), 3151–3157. 24018423 10.1194/jlr.M042556 PMC3793619

[tjp70812-bib-0017] Haga, T. (2013). Molecular properties of muscarinic acetylcholine receptors. Proceedings of the Japan Academy, Series B: Physical and Biological Sciences, 89(6), 226–256. 23759942 10.2183/pjab.89.226 PMC3749793

[tjp70812-bib-0018] Jain, T. , Wager‐Miller, J. , Mackie, K. , & Straiker, A. (2013). Diacylglycerol lipasealpha (DAGLalpha) and DAGLbeta cooperatively regulate the production of 2‐arachidonoyl glycerol in autaptic hippocampal neurons. Molecular Pharmacology, 84(2), 296–302. 23748223 10.1124/mol.113.085217 PMC3716324

[tjp70812-bib-0019] Jayamanne, A. , Greenwood, R. , Mitchell, V. A. , Aslan, S. , Piomelli, D. , & Vaughan, C. W. (2006). Actions of the FAAH inhibitor URB597 in neuropathic and inflammatory chronic pain models. British Journal of Pharmacology, 147(3), 281–288. 16331291 10.1038/sj.bjp.0706510 PMC1751298

[tjp70812-bib-0020] Jung, K. M. , Sepers, M. , Henstridge, C. M. , Lassalle, O. , Neuhofer, D. , Martin, H. , Ginger, M. , Frick, A. , Dipatrizio, N. V. , Mackie, K. , Katona, I. , Piomelli, D. , & Manzoni, O. J. (2012). Uncoupling of the endocannabinoid signalling complex in a mouse model of fragile X syndrome. Nature Communications, 3(1), 1080. 10.1038/ncomms2045 PMC3657999 23011134

[tjp70812-bib-0021] Kano, M. , Ohno‐Shosaku, T. , Hashimotodani, Y. , Uchigashima, M. , & Watanabe, M. (2009). Endocannabinoid‐mediated control of synaptic transmission. Physiological Reviews, 89(1), 309–380. 19126760 10.1152/physrev.00019.2008

[tjp70812-bib-0022] Leishman, E. , Mackie, K. , Luquet, S. , & Bradshaw, H. B. (2016). Lipidomics profile of a NAPE‐PLD KO mouse provides evidence of a broader role of this enzyme in lipid metabolism in the brain. Biochimica Et Biophysica Acta, 1861(6), 491–500. 26956082 10.1016/j.bbalip.2016.03.003 PMC4909477

[tjp70812-bib-0023] Lemullois, M. , Rossignol, B. , & Mauduit, P. (1996). Immunolocalization of myoepithelial cells in isolated acini of rat exorbital lacrimal gland: Cellular distribution of muscarinic receptors. Biologie Cellulaire, 86(2‐3), 175–181. 10.1016/0248-4900(96)84782-4 8893507

[tjp70812-bib-0024] Leung, D. , Saghatelian, A. , Simon, G. M. , & Cravatt, B. F. (2006). Inactivation of N‐acyl phosphatidylethanolamine phospholipase D reveals multiple mechanisms for the biosynthesis of endocannabinoids. Biochemistry, 45(15), 4720–4726. 16605240 10.1021/bi060163l PMC1538545

[tjp70812-bib-0025] Ligresti, A. , Morera, E. , Van Der Stelt, M. , Monory, K. , Lutz, B. , Ortar, G. , & Di Marzo, V. (2004). Further evidence for the existence of a specific process for the membrane transport of anandamide. Biochemical Journal, 380(1), 265–272. 14969584 10.1042/BJ20031812 PMC1224156

[tjp70812-bib-0026] Magotti, P. , Bauer, I. , Igarashi, M. , Babagoli, M. , Marotta, R. , Piomelli, D. , & Garau, G. (2015). Structure of human N‐acylphosphatidylethanolamine‐hydrolyzing phospholipase D: Regulation of fatty acid ethanolamide biosynthesis by bile acids. Structure, 23(3), 598–604. 25684574 10.1016/j.str.2014.12.018 PMC4351732

[tjp70812-bib-0027] Margheritis, E. , Castellani, B. , Magotti, P. , Peruzzi, S. , Romeo, E. , Natali, F. , Mostarda, S. , Gioiello, A. , Piomelli, D. , & Garau, G. (2016). Bile acid recognition by NAPE‐PLD. American Chemical Society Chemical Biology, 11(10), 2908–2914. 27571266 10.1021/acschembio.6b00624 PMC5074845

[tjp70812-bib-0028] Mitchelson, F. (2012). Muscarinic receptor agonists and antagonists: Effects on ocular function. Handbook of Experimental Pharmacology, (pp. 263–298). Springer. 10.1007/978-3-642-23274-9_12 22222703

[tjp70812-bib-0029] Mock, E. D. , Mustafa, M. , Gunduz‐Cinar, O. , Cinar, R. , Petrie, G. N. , Kantae, V. , Di, X. , Ogasawara, D. , Varga, Z. V. , Paloczi, J. , Miliano, C. , Donvito, G. , van Esbroeck, A. C. M. , van der Gracht, A. M. F. , Kotsogianni, I. , Park, J. K. , Martella, A. , van der Wel, T. , Soethoudt, M. , … van der Stelt, M. (2020). Discovery of a NAPE‐PLD inhibitor that modulates emotional behavior in mice. Nature Chemical Biology, 16(6), 667–675. 32393901 10.1038/s41589-020-0528-7 PMC7468568

[tjp70812-bib-0030] Munro, S. , Thomas, K. L. , & Abu‐Shaar, M. (1993). Molecular characterization of a peripheral receptor for cannabinoids. Nature, 365(6441), 61–65. 7689702 10.1038/365061a0

[tjp70812-bib-0031] Murataeva, N. , Mattox, S. , Lemieux, J. , Griffis, J. , Yust, K. , Du, W. , Heinbockel, T. , & Straiker, A. (2024). Cannabinoid regulation of sex‐dependent murine odorant‐stimulated salivation. Scientific Reports, 14(1), 26720. 39496764 10.1038/s41598-024-77761-4 PMC11535013

[tjp70812-bib-0032] Murataeva, N. , Straiker, A. , & Mackie, K. (2014). Parsing the players: 2‐arachidonoylglycerol synthesis and degradation in the CNS. British Journal of Pharmacology, 171(6), 1379–1391. 24102242 10.1111/bph.12411 PMC3954479

[tjp70812-bib-0033] Murataeva, N. , Youkilis, J. , Rao, Y. , & Straiker, A. (2026). Don't sweat it: Cannabinoid CB1 receptors reduce sweating in a mouse model. Federation of American Societies for Experimental Biology Journal, 40(12), e72051. 42287607 10.1096/fj.202601143R PMC13264398

[tjp70812-bib-0034] Murataeva, N. , Yust, K. , Mattox, S. , Du, W. , & Straiker, A. (2024). A sex‐dependent cannabinoid CB1 receptor role in circadian tearing of the mouse. Investigative Ophthalmology & Visual Science, 65(14), 10. 10.1167/iovs.65.14.10 PMC11622162 39630463

[tjp70812-bib-0035] Natarajan, V. , Schmid, P. C. , Reddy, P. V. , Zuzarte‐Augustin, M. L. , & Schmid, H. H. O. (1983). Biosynthesis of N‐acylethanolamine phospholipids by dog brain preparations. Journal of Neurochemistry, 41(5), 1303–1312. 6619867 10.1111/j.1471-4159.1983.tb00825.x

[tjp70812-bib-0036] Nyilas, R. , Dudok, B. , Urbán, G. M. , Mackie, K. , Watanabe, M. , Cravatt, B. F. , Freund, T. F. , & Katona, I. (2008). Enzymatic machinery for endocannabinoid biosynthesis associated with calcium stores in glutamatergic axon terminals. Journal of Neuroscience, 28(5), 1058–1063. 18234884 10.1523/JNEUROSCI.5102-07.2008 PMC6671412

[tjp70812-bib-0037] Ohno‐Shosaku, T. , Shosaku, J. , Tsubokawa, H. , & Kano, M. (2002). Cooperative endocannabinoid production by neuronal depolarization and group I metabotropic glutamate receptor activation. European Journal of Neuroscience, 15(6), 953–961. 11918654 10.1046/j.1460-9568.2002.01929.x

[tjp70812-bib-0038] Okamoto, Y. , Morishita, J. , Tsuboi, K. , Tonai, T. , & Ueda, N. (2004). Molecular characterization of a phospholipase D generating anandamide and its congeners. Journal of Biological Chemistry, 279(7), 5298–5305. 14634025 10.1074/jbc.M306642200

[tjp70812-bib-0039] Okamoto, Y. , Wang, J. , Morishita, J. , & Ueda, N. (2007). Biosynthetic pathways of the endocannabinoid anandamide. Chemistry and Biodiversity, 4(8), 1842–1857. 17712822 10.1002/cbdv.200790155

[tjp70812-bib-0040] Schiavone, A. , & Brambilla, A. (1991). Muscarinic M3 receptors mediate secretion from sweat glands in the rat. Pharmacological Research, 23(3), 233–239. 2068048 10.1016/s1043-6618(05)80082-9

[tjp70812-bib-0041] Schmid, P. C. , Reddy, P. V. , Natarajan, V. , & Schmid, H. H. (1983). Metabolism of N‐acylethanolamine phospholipids by a mammalian phosphodiesterase of the phospholipase D type. Journal of Biological Chemistry, 258(15), 9302–9306. 6308001

[tjp70812-bib-0042] Shonesy, B. C. , Stephenson, J. R. , Marks, C. R. , & Colbran, R. J. (2020). Cyclic AMP‐dependent protein kinase and D1 dopamine receptors regulate diacylglycerol lipase‐alpha and synaptic 2‐arachidonoyl glycerol signaling. Journal of Neurochemistry, 153(3), 334–345. 31985073 10.1111/jnc.14972 PMC7367494

[tjp70812-bib-0043] Simon, G. M. , & Cravatt, B. F. (2006). Endocannabinoid biosynthesis proceeding through glycerophospho‐N‐acyl ethanolamine and a role for alpha /beta hydrolase 4 in this pathway. Journal of Biological Chemistry, 281(36), 26465–24672. 16818490 10.1074/jbc.M604660200

[tjp70812-bib-0044] Singh, S. , Sarroza, D. , English, A. , McGrory, M. , Dong, A. , Zweifel, L. , Land, B. B. , Li, Y. , Bruchas, M. R. , & Stella, N. (2023). Pharmacological characterization of the endocannabinoid sensor GRAB_eCB2.0_ . Cannabis and Cannabinoid Research, 9(5), 1250–1266. 38064488 10.1089/can.2023.0036 PMC11535446

[tjp70812-bib-0045] Smart, D. , Gunthorpe, M. J. , Jerman, J. C. , Nasir, S. , Gray, J. , Muir, A. I. , Chambers, J. K. , Randall, A. D. , & Davis, J. B. (2000). The endogenous lipid anandamide is a full agonist at the human vanilloid receptor (hVR1). British Journal of Pharmacology, 129(2), 227–230. 10694225 10.1038/sj.bjp.0703050 PMC1571834

[tjp70812-bib-0046] Straiker, A. , & Mackie, K. (2005). Depolarization‐induced suppression of excitation in murine autaptic hippocampal neurones. The Journal of Physiology, 569(2), 501–517. 16179366 10.1113/jphysiol.2005.091918 PMC1464237

[tjp70812-bib-0047] Straiker, A. , & Mackie, K. (2007). Metabotropic suppression of excitation in murine autaptic hippocampal neurons. The Journal of Physiology, 578(3), 773–785. 17110416 10.1113/jphysiol.2006.117499 PMC2151347

[tjp70812-bib-0048] Sugiura, T. , Kondo, S. , Sukagawa, A. , Tonegawa, T. , Nakane, S. , Yamashita, A. , Ishima, Y. , & Waku, K. (1996). Transacylase‐mediated and phosphodiesterase‐mediated synthesis of N‐arachidonoylethanolamine, an endogenous cannabinoid‐receptor ligand, in rat brain microsomes. Comparison with synthesis from free arachidonic acid and ethanolamine. European Journal of Biochemistry, 240(1), 53–62. 8797835 10.1111/j.1432-1033.1996.0053h.x

[tjp70812-bib-0049] Thayer, A. , Murataeva, N. , Delcroix, V. , Wager‐Miller, J. , Makarenkova, H. P. , & Straiker, A. (2020). THC regulates tearing via cannabinoid CB1 receptors. Investigative Ophthalmology & Visual Science, 61(10), 48. 10.1167/iovs.61.10.48 PMC7452851 32852544

[tjp70812-bib-0050] Tsuboi, K. , Sun, Y. X. , Okamoto, Y. , Araki, N. , Tonai, T. , & Ueda, N. (2005). Molecular characterization of N‐acylethanolamine‐hydrolyzing acid amidase, a novel member of the choloylglycine hydrolase family with structural and functional similarity to acid ceramidase. Journal of Biological Chemistry, 280(12), 11082–11092. 15655246 10.1074/jbc.M413473200

[tjp70812-bib-0051] Ueda, N. , Liu, Q. , & Yamanaka, K. (2001). Marked activation of the N‐acylphosphatidylethanolamine‐hydrolyzing phosphodiesterase by divalent cations. Biochimica Et Biophysica Acta, 1532(1‐2), 121–127. 11420181 10.1016/s1388-1981(01)00120-2

[tjp70812-bib-0052] Ueda, N. , Okamoto, Y. , & Tsuboi, K. (2005). Endocannabinoid‐related enzymes as drug targets with special reference to N‐acylphosphatidylethanolamine‐hydrolyzing phospholipase D. Current Medicinal Chemistry, 12(12), 1413–1422. 15974992 10.2174/0929867054020918

[tjp70812-bib-0053] Urso, A. , D'Ovidio, F. , Xu, D. , Emala, C. W. , Bunnett, N. W. , & Perez‐Zoghbi, J. F. (2020). Bile acids inhibit cholinergic constriction in proximal and peripheral airways from humans and rodents. American Journal of Physiology‐Lung Cellular and Molecular Physiology, 318(2), L264–L275. 31800261 10.1152/ajplung.00242.2019 PMC7474253

[tjp70812-bib-0054] van der Stelt, M. , & Di Marzo, V. (2005). Anandamide as an intracellular messenger regulating ion channel activity. Prostaglandins & Other Lipid Mediators, 77(1‐4), 111–122. 16099396 10.1016/j.prostaglandins.2004.09.007

[tjp70812-bib-0055] van der Stelt, M. , Trevisani, M. , Vellani, V. , De Petrocellis, L. , Schiano Moriello, A. , Campi, B. , McNaughton, P. , Geppetti, P. , & Di Marzo, V. (2005). Anandamide acts as an intracellular messenger amplifying Ca^2+^ influx via TRPV1 channels. European Molecular Biology Organization Journal, 24(17), 3026–3037. 10.1038/sj.emboj.7600784 PMC1201361 16107881

[tjp70812-bib-0056] Wang, J. , Okamoto, Y. , Morishita, J. , Tsuboi, K. , Miyatake, A. , & Ueda, N. (2006). Functional analysis of the purified anandamide‐generating phospholipase D as a member of the metallo‐beta‐lactamase family. Journal of Biological Chemistry, 281(18), 12325–12335. 16527816 10.1074/jbc.M512359200

