# Supplementary material for: N‐Arachidonoyl‐phosphatidylethanolamide phospholipase D activation by muscarinic receptors
Source: J Physiol. 2026 Sep 2;604(18):7721–38. doi: 10.1113/JP291508 (PMC13577693; doi:10.1113/JP291508)
Supplement: Supplementary file 1 [file TJP-604-7721-s001.pdf]

The Journal of Physiology

NAPE-PLD activation by muscarinic receptors

Natalia Murataeva, Connor Schmitt, Elyssa Hyman, Jim Wager-Miller, Irene Matthews, Ruyi Cai, Shangxuan Cai, Yulong Li, Ken Mackie, and Alex Straiker
DOI: 10.1113/JP291508

Corresponding author(s): Alex Straiker (straiker@iu.edu)

The referees have opted to remain anonymous.

Review Timeline:

Submission Date:	05-Apr-2026
Editorial Decision:	22-Jun-2026
Revision Received:	02-Jul-2026
Accepted:	09-Aug-2026

Senior Editor: Jing-Ning Zhu

Reviewing Editor: Jing-Ning Zhu

Transaction Report:

(Note: With the exception of the correction of typographical or spelling errors that could be a source of ambiguity, letters and reports are not edited. Depending on transfer agreements, referee reports obtained elsewhere may or may not be included in this compilation. Referee reports are anonymous unless the Referee chooses to sign their reports.)

Re: JP-RP-2026-291508 "**NAPE-PLD activation by muscarinic receptors** " by Natalia Murataeva, Connor Schmitt, Elyssa Hyman, Jim Wager-Miller, Irene Matthews, Ruyi Cai, Shangxuan Cai, Yulong Li, Ken Mackie, and Alex Straiker

Dear Dr Straiker,

Thank you for submitting your manuscript to The Journal of Physiology. It has been assessed by a Reviewing Editor and by 2 expert referees and we are pleased to tell you that it is potentially acceptable for publication following satisfactory major revision.

Please advise your co-authors of this decision as soon as possible.

The referee reports are copied at the end of this email.

Please address all the points raised and incorporate all requested revisions or explain in your Response to Referees why a change has not been made. We hope you will find the comments helpful and that you will be able to return your revised manuscript within 2 months. If your article is NOT for a Special Issue, you may have 9 months to revise. If you require an extension, please contact journal staff: jp@physoc.org. Please note that this letter does not constitute a guarantee for acceptance of your revised manuscript.

Your revised manuscript should be submitted online using the link in your Author Tasks: Link Not Available. This link is accessible via your account as Corresponding Author; it is not available to your co-authors. If this presents a problem, please contact journal staff (jp@physoc.org). Image files from the previous version are retained on the system. Please ensure you replace or remove any files that are being revised.

If you do not wish to submit a revised version of your manuscript, you must inform our journal staff (jp@physoc.org) or reply to this email to request withdrawal. Please note that a manuscript must be formally withdrawn from the peer review process at one journal before it may be submitted to another journal.

TRANSPARENT PEER REVIEW POLICY: To improve the transparency of its peer review process, The Journal of Physiology publishes online as supporting information the peer review history of all articles accepted for publication. Readers will have access to decision letters, including Editors' comments and referee reports, for each version of the manuscript, as well as any author responses to peer review comments. Referees can decide whether or not they wish to be named on the peer review history document.

ABSTRACT FIGURES: Authors are expected to use The Journal's premium BioRender account to create/redraw their Abstract Figures. Information on how to access this account is here: https://physoc.onlinelibrary.wiley.com/journal/14697793/biorender-access.

This will enable Authors to create and download high-resolution figures. If authors have used the free BioRender service, they can use the instructions provided in the link above to download a high-resolution version suitable for publication.

The link provided should only be used for the purposes of this submission. Authors will be charged for figures created on this account if they are not related to this manuscript submission.

LANGUAGE EDITING AND SUPPORT FOR PUBLICATION: If you would like help with English language editing, or other article preparation support, Wiley Editing Services offers expert help, including English Language Editing, as well as translation, manuscript formatting, and figure formatting at www.wileyauthors.com/eeo/preparation. You can also find resources for Preparing Your Article for general guidance about writing and preparing your manuscript at www.wileyauthors.com/eeo/prepresources.

RIGOUR AND REPRODUCIBILITY: We recommend authors consult The Journal's Rigour and Reproducibility requirements to ensure that their manuscript is suitable for publication: https://physoc.onlinelibrary.wiley.com/pb-assets/hub-assets/physoc/documents/TJP-Rigour-and-Reproducibility-Requirements.pdf

REVISION CHECKLIST:

Check that your Methods section conforms to journal policy: https://jp.msubmit.net/cgi-bin/main.plex?form_type=display_requirements#methods.

Check that data presented conforms to the statistics policy: https://jp.msubmit.net/cgi-bin/main.plex?form_type=display_requirements#statistics.

Upload a full Response to Referees file. To create your 'Response to Referees': copy all the reports, including any comments from the Senior and Reviewing Editors, into a Microsoft Word, or similar, file and respond to each point, using font or background colour to distinguish comments and responses and upload as the required file type.

Please upload two versions of your manuscript text: one with all relevant changes highlighted and one clean version with no changes tracked. The manuscript file should include all tables and figure legends, but each figure/graph should be uploaded as separate, high-resolution files.

You may also upload:

- 'Potential Cover Art' for consideration as the issue's cover image
- Appropriate Supporting Information (Video, audio or data set: see https://jp.msubmit.net/cgi-bin/main.plex?form_type=display_requirements#supp).

We look forward to receiving your revised submission.

If you have any queries, please reply to this email and we will be pleased to advise.

Yours sincerely,

Jing-Ning Zhu
Senior Editor
The Journal of Physiology

REQUIRED ITEMS

- Author photo and profile. First or joint first authors are asked to provide a short biography (no more than 100 words for one author or 150 words in total for joint first authors) and a portrait photograph. These should be uploaded and clearly labelled together in a Word document with the revised version of the manuscript. See Information for Authors for further details.

- Research must comply with The Journal's policies regarding animal experiments (https://physoc.onlinelibrary.wiley.com/hub/animal-experiments) and adherence to these policies must be stated in the manuscript. The Methods section must contain:
(1) a statement about housing,
(2) details of the anaesthetic regime, including the dose.

- The reference list must be in alphabetical order, rather than numbered, to comply with our Journal format.

- Your manuscript must include a complete Additional Information section, including competing interests, and author contributions.

- Please upload separate high-quality figure files via the submission form.

- Please ensure that any tables are editable and in Word format, and wherever possible, embedded in the article file itself.

- Please ensure that the Article File you upload is a Word file.

- Papers must comply with the Statistics Policy: https://jp.msubmit.net/cgi-bin/main.plex?form_type=display_requirements#statistics.

In summary:

- The most appropriate summary statistic (e.g. mean or median and standard deviation) must be used. Standard Error of the Mean (SEM) alone is not permitted.

- A Data Availability Statement is required for all papers reporting original data. This must be in the Additional Information section of the manuscript itself. It must have the paragraph heading 'Data Availability Statement'. All data supporting the results in the paper must be either: in the paper itself; uploaded as Supporting Information for Online Publication; or archived in an appropriate public repository. The statement needs to describe the availability or the absence of shared data. Authors must include in their statement: a link to the repository they have used, or a statement that it is available as Supporting Information; reference the data in the appropriate sections(s) of their manuscript; and cite the data they have shared in the References section. Whenever possible, the scripts and other artefacts used to generate the analyses presented in the paper should also be publicly archived. If sharing data compromises ethical standards or legal requirements then authors are not expected to share it, but must note this in their statement. For more information, see our Statistics Policy.

- Please include an Abstract Figure file and an Abstract Figure legend. An appropriate figure legend, which should not exceed 150 words in length, should be included in the main manuscript file. The Abstract Figure is a piece of artwork designed to give readers an immediate understanding of the research and should summarise the main conclusions. If possible, the image should be easily 'readable' from left to right or top to bottom. It should show the physiological relevance of the manuscript so readers can assess the importance and content of its findings. Abstract Figures should not merely recapitulate other figures in the manuscript. Please try to keep the diagram as simple as possible and without superfluous information that may distract from the main conclusion(s). Abstract Figures must be provided by authors no later than the revised manuscript stage and should be uploaded as a separate file during online submission labelled as File Type 'Abstract Figure'. Please also ensure that you include the figure legend in the main article file. All Abstract Figures should be created using BioRender. Authors should use The Journal's premium BioRender account to export high-resolution images. Details on how to use and access the premium account are included as part of this email.

- Authors are required to fully disclose the use of any Generative AI (GAI) tools used in the preparation of their manuscript. This disclosure should also be included as a statement alongside the Additional Information and Data Availability statements towards the end of the article under the heading 'Generative AI Statement', and include the name(s), model and version of AI technology used, and exact details on the purpose for, and methods of, its use.

If no AI tool has been used, this should also be specifically stated within the manuscript.

EDITOR COMMENTS

Senior Editor:

Thank you for submitting your manuscript to The Journal of Physiology. Both reviewers find the topic timely and the study well conducted, but they raise several issues that must be addressed before we can proceed. In brief, Reviewer 1 raises points regarding methodological details, figure consistency, and sex differences. Reviewer 2 raises more substantive concerns, including the need to update references on NAPE-PLD, address the technical limitation posed by endogenous NAPE-PLD expression in HEK293 cells, revise figure presentations, and moderate speculative claims about general GPCR activation. We encourage you to carefully address all points raised in your revision. Please submit a revised manuscript accompanied by a point-by-point response. I look forward to receiving your revision.

REFEREE COMMENTS

Referee #1:

Review to Murataeva et al.

This is a very solid and clear dissection of the eCB signaling system in the submandibular salivary gland, addressing fundamental questions on the differential functions of AEA and 2-AG, but also shedding light on the observation, why cannabis use may lead to dry mouth and eyes. The paper is excellently structured, data presentation is of equal quality. The conclusions are well based on the experimental observations. Thus, there are only minor issues which are suggested below:

In the method part, it is said that both males and females were used. In some figures, specifically one sex is mentioned in the legend. Question: are there sex differences reported in humans regarding dry mouth and eyes? It is suggested to mention whether or not sex differences were analyzed in this study.

1) Please give references to the various eCB sensors in the material part.

2) Page 14, line 1: this sentence is not clear.

3) Figure 6: please use in B the same presentation as in A, A1, and A2.

4) Figure 7: Here, rat glands were isolated, but the procedure is not described in the method part.

5) Page 16: It might be helpful to describe in more details how gland sections were placed on the sensor cell layer. Does the section stick to the bottom, or is there a "fixation" needed?

Referee #2:

In this work Straiker & co-authors have investigated the activation of membrane phospholipase NAPE-PLD by muscarinic M3 receptors. They provide evidence using natively-expressed M3 HEK293 cells co-transfected with NAPE-PLD and GRABeCB sensors. Furthermore, they have explored the co-localization of NAPE PLD and M3 proteins in myoepithelial cells using immunohistochemistry, suggesting that M3R can stimulate indirectly NAPE-PLD to produce AEA. They finally hypothesized that the GCPR activation may be a general mechanism to induce the synthesis of AEA by NAPE-PLD.

Given the enormous medical and physiological interest that the therapeutic target NAPE-PLD has generated in recent years, the topic is undoubtedly of great interest to readers of the Journal of Physiology and the scientific community in the field.

Anyhow, some important clarifications are necessary to further support and enhance the findings of this work.

Here are my comments and suggestions:

1. As reported in the Abstract (lines 5-8) and in the Introduction (lines 8-17), authors aim to explore (1) the synthesis of AEA by NAPE-PLD and (2) a way by which this key enzyme of the ECS may be activated.

Anyhow, in recent years some innovative scientific works have examined in detail fundamental aspects of these points. In particular, the zinc-mediated catalytic mechanism of NAPE-PLD have been structurally and functionally unveiled, including the relevant physiological discovery that NAPE-PLD is modulated by bile acids [e.g., Structure 2015].

Given the relevance of the possible contribution of M3 in stimulating the production of AEA by NAPE-PLD, it is certainly appropriate to mention and comment on results of these investigations in the abstract/introduction, discussion and Figure 1).

Of note, the old reference (7) might be not appropiate for the sentance (line 6) "...NAPE-PLD, a zinc metallohydrolase

(7)" because it mentions zinc as inhibitory ion.

2. The authors report in the manuscript that "muscarinic M3 receptors are GPCRs natively expressed in HEK293 cells" and

have used NAPE-PLD transfected HEK293 cells for the experiments. Anyhow, HEK293 cells are also known to express NAPE-PLD natively [e.g., Guo et al J LIpid Res 2013]. Thus, authors have performed experiments only by overexpressing NAPE-PLD but not M3 on cells, which naturally express both M3 and NAPE-PLD. This greatly complicates the interpretation of their results.

Hence, to validate their results, the Authors should confirm the results on HEK293 cells not overexpressing NAPE-PLD and possibly on HEK293 cells overexpressing both proteins. Moreover, the authors should comment extensively on this methodological aspect in the results and discussion, expressing the technical limitations of their findings.

3. The images A1 and A2 of Fig. 6 and images B and D of Fig. 7 have to show the specific colors of the corresponding image in A or A and B, respectively.

4. Previous results show that bile acids inhibit the acetylcholine-induced inositol phosphate synthesis in human cells overexpressing the muscarinic M3 receptor [e.g., Am J Physiol Lung Cell Mol Physiol Structur2020]. As bile acids bind and structurally stabilize and modulate the activity of NAPE-PLD, the authors should consider their findings in relation with these important results in the discussion.

5. Both M3 and NAPE-PLD are liked with hypertension [Alvares-Lopez et al. Canadian Journal of Cardiology 2019; Chiarugi et al Cell Chem Biol 2025]. Authors should discuss this important aspect in their manuscript to stress the functional and physiological relevance of their results.

6. While previous results undoubtedly support the connection between muscarinic receptors and the ECS [e.g., Foster et al Neuron 2016], the hypothesis that the activation of GPCRs might generally stimulate AEA production is somewhat speculative (e.g., "Gq-coupled GPCR activation of NAPE PLD may be a general mechanism to induce anandamide synthesis"). Considereing the differennces among distinct GCPRs, the results of this study do not support this view.

I would suggest the Authors to reserve this hypothesis for future studies in the field.

Conversely, it may be helpful for a general reader to have a clear understanding of the specific distribution and physiological function of different muscarinic receptors (starting from M3 and M4) and how these individually may contribute to the physiological function of the ECS centrally and peripherally (e.g. injury, inflammation).

END OF COMMENTS

We thank the reviewers for their close attention to our manuscript and for their constructive suggestions for improvements. We believe that the reviewer comments have helped to improve the manuscript. Below please find a point-by-point reply to reviewer concerns below. In some instances, we have abbreviated the reviewer comment to focus on the requested changes. We have also updated the manuscript to comply with Journal of Physiology formatting requirements. We have also added a 1st author bio and an Abstract Figure.

Referee #1:

In the method part, it is said that both males and females were used. In some figures, specifically one sex is mentioned in the legend.

A: Thank you for pointing this out – it needed clarification. We tested salivary glands from both males and females, but for lacrimal glands we only made use of females because we have seen a sex-dependence of cannabinoid regulation of tearing. We have clarified the language in the methods section on this point (chiefly page 9, lines 18-19).

Question: are there sex differences reported in humans regarding dry mouth and eyes? It is suggested to mention whether or not sex differences were analyzed in this study.
A: There is certainly a sex-difference in the incidence of dry mouth and dry eye. For instance, 80% of those suffering from Sjogren's Syndrome an inflammatory disease that affects both are female. We have expanded on this in the methods section (page 10, lines 1-5)

1) Please give references to the various eCB sensors in the material part.
A: We have added references regarding the GRAB sensors.

2) Page 14, line 1: this sentence is not clear.
A: We assume this relates to the sentence starting with "We included rat…" and have revised this to make it clearer.

3) Figure 6: please use in B the same presentation as in A, A1, and A2.

A: Figure 6 has been updated as requested.

4) Figure 7: Here, rat glands were isolated, but the procedure is not described in the method part.
A: This has been updated.

5) Page 16: It might be helpful to describe in more details how gland sections were placed on the sensor cell layer. Does the section stick to the bottom, or is there a "fixation" needed?

A: The tissue sections weren't held down. We have added details about this to that section.

Referee #2:

1. As reported in the Abstract (lines 5-8) and in the Introduction (lines 8-17), authors aim to explore (1) the synthesis of AEA by NAPE-PLD and (2) a way by which this key enzyme of the ECS may be activated.

Anyhow, in recent years some innovative scientific works have examined in detail fundamental aspects of these points. In particular, the zinc-mediated catalytic mechanism of NAPE-PLD have been structurally and functionally unveiled, including the relevant physiological discovery that NAPE-PLD is modulated by bile acids [e.g., Structure 2015].

Given the relevance of the possible contribution of M3 in stimulating the production of AEA by NAPE-PLD, it is certainly appropriate to mention and comment on results of these investigations in the abstract/introduction, discussion and Figure 1). Of note, the old reference (7) might be not appropriate for the sentence (line 6) "...NAPE-PLD, a zinc metallohydrolase (7)" because it mentions zinc as inhibitory ion.

A: We have added details/discussion on regulation of NAPE-PLD by bile acids as well as the importance of zinc. Discussion of this may be found on P26, lines 1-7. Please note that we have changed the format of the references to JPhys style, so they now appear in alphabetical order rather than numerical. Regarding the Schmid 1983 reference, we believe that this early work made an important early contribution and would prefer to include an additional reference rather than replace it outright. Moreover, subsequent experiments by Dr. Ueda's group reinforced the idea that Zinc is essential, but that exogenous zinc inhibits enzymatic activity (Ueda 2005). We hope that the reviewer will find it acceptable if we do not add modulation of NAPE-PLD by zinc or bile salts to Figure 1 since the figure is already dense and we are concerned that by adding additional details the figure will become difficult for readers to easily comprehend.

2. The authors report in the manuscript that "muscarinic M3 receptors are GPCRs natively expressed in HEK293 cells" and have used NAPE-PLD transfected HEK293 cells for the experiments. Anyhow, HEK293 cells are also known to express NAPE-PLD natively [e.g., Guo et al J LIpid Res 2013]. Thus, authors have performed experiments only by overexpressing NAPE-PLD but not M3 on cells, which naturally express both

M3 and NAPE-PLD. This greatly complicates the interpretation of their results.

Hence, to validate their results, the Authors should confirm the results on HEK293 cells not overexpressing NAPE-PLD and possibly on HEK293 cells overexpressing both proteins. Moreover, the authors should comment extensively on this methodological aspect in the results and discussion, expressing the technical limitations of their findings.

A: This is an important point. We noted in the results section (P11, Line 3) that the responses to oxo-M are absent in cells transfected with only the sensor. We conclude from this that basal NAPE-PLD levels are insufficient to produce a measurable response. We have added mention of this question to the discussion including the Guo et al. 2013 reference (last lines of P25).

3. The images A1 and A2 of Fig. 6 and images B and D of Fig. 7 have to show the specific colors of the corresponding image in A or A and B, respectively.
A: With all due respect, white is superior for the single-channel images. The human retina is optimized for green, meaning that red and especially blue images are suboptimal.

4. Previous results show that bile acids inhibit the acetylcholine-induced inositol phosphate synthesis in human cells overexpressing the muscarinic M3 receptor [e.g., Am J Physiol Lung Cell Mol Physiol Structure 2020]. As bile acids bind and structurally stabilize and modulate the activity of NAPE-PLD, the authors should consider their findings in relation with these important results in the discussion
A: We are concerned that expansion on this topic in the discussion will be seen as overly speculative. While bile acid interactions are undoubtedly relevant to NAPE-PLD activity and are now mentioned in the manuscript in several places, it is unclear how we can introduce the question of bile acid modulation of muscarinic signaling without making that section too diffuse. In the course of reading up on this topic we encountered a paper proposing that bile acids directly inhibit muscarinic receptors, which would further complicate the picture. This subject merits further research and perhaps discussion in a review article.

5. Both M3 and NAPE-PLD are liked with hypertension [Alvares-Lopez et al. Canadian Journal of Cardiology 2019; Chiarugi et al Cell Chem Biol 2025]. Authors should discuss this important aspect in their manuscript to stress the functional and physiological relevance of their results.
A: We have added a section offering several examples of potential functional and physiological consequences of M3/NAPE-PLD signaling, including the Chiarugi paper suggested by the reviewer. Regrettably we have been unable to identify the Alvares-Lopez 2019 paper mentioned by the reviewer. (latter half of page 28)

6. While previous results undoubtedly support the connection between muscarinic receptors and the ECS [e.g., Foster et al Neuron 2016], the hypothesis that the activation of GPCRs might generally stimulate AEA production is somewhat speculative (e.g., "Gq-coupled GPCR activation of NAPE PLD may be a general mechanism to induce anandamide synthesis"). Considering the differences among distinct GCPRs, the results of this study do not support this view.

I would suggest the Authors to reserve this hypothesis for future studies in the field.
A: We have revised this statement to be more conservative – it now is restricted to muscarinic stimulation.

Conversely, it may be helpful for a general reader to have a clear understanding of the specific distribution and physiological function of different muscarinic receptors (starting from M3 and M4) and how these individually may contribute to the physiological function of the ECS centrally and peripherally (e.g. injury, inflammation).
A: We agree that the manuscript would benefit from some discussion of muscarinic receptor subtype contributions though an anatomical survey of muscarinic receptor distribution is probably outside the scope of this manuscript. There are five muscarinic receptors, three of which (M1, M3 and M5) are Gq-coupled (M2 and M4 are Gi/o-coupled). Taking the example of lacrimation, there is evidence for contribution of M1 and M3 receptors but M3 appears to underlie the bulk of cholinergic regulation of tearing. We have elaborated on this in the discussion (P28, lines 7-13)

Dear Dr Straiker,

Re: JP-RP-2026-291508R1 "**NAPE-PLD activation by muscarinic receptors** " by Natalia Murataeva, Connor Schmitt, Elyssa Hyman, Jim Wager-Miller, Irene Matthews, Ruyi Cai, Shangxuan Cai, Yulong Li, Ken Mackie, and Alex Straiker

We are pleased to tell you that your paper has been accepted for publication in The Journal of Physiology.

The last Word (or similar) version of the manuscript provided will be used by the Production Editor to prepare your proof. When this is ready you will receive an email containing a link to Wiley's Online Proofing System. The proof should be thoroughly checked and corrected as promptly as possible.

Authors should note that it is too late at this point to offer corrections prior to proofreading. Major corrections at proof stage, such as changes to figures, will be referred to the Editors for approval before they can be incorporated. Only minor changes, such as to style and consistency, should be made at proof stage. Changes that need to be made after proof stage will usually require a formal correction notice.

All queries at proof stage should be sent to: TJP@wiley.com.

Following copyediting and typesetting the accepted version will be published online.

Yours sincerely,

Jing-Ning Zhu
Senior Editor
The Journal of Physiology

IMPORTANT POINTS TO NOTE FOLLOWING ACCEPTANCE OF YOUR PAPER:

• IMPORTANT NOTICE ABOUT OPEN ACCESS: To assist authors whose funding agencies mandate immediate public access to published research findings, The Journal of Physiology allows authors to pay an Open Access (OA) fee to have their papers made freely available immediately on publication.

The Corresponding Author will receive an email from Wiley with details on how to register or log in to Wiley Authors where you will be able to place an order.

• You can check if your funder or institution has a Wiley Open Access Account here:
https://authors.wiley.com/author-resources/Journal-Authors/open-access/author-compliance-tool.html

MEMBER DISCOUNT: Corresponding authors who are Members of the Physiological Society are eligible for a 20% discount on the cost of the Article Processing Charge (APC). To claim this discount, the Corresponding Author will need to log in to the Physiological Society Membership portal and retrieve the discount code (https://www.physoc.org/journals-and-media/journals/journal-discount-for-members/). This code should then be quoted in the billing section of Wiley Authors when selecting the open access option.

• TRANSPARENT PEER REVIEW POLICY: To improve the transparency of its peer review process, The Journal of Physiology publishes online as supporting information the peer review history of all articles accepted for publication. Readers will have access to decision letters, including Editors' comments and referee reports, for each version of the manuscript as well as any author responses to peer review comments. Referees can decide whether or not they wish to be named on the peer review history document.

• You can help your research get the attention it deserves! Check out Wiley's free Promotion Guide for best-practice recommendations for promoting your work at: www.wileyauthors.com/eeo/guide. You can learn more about Wiley Editing Services which offers professional video, design, and writing services to create shareable video abstracts, infographics, conference posters, lay summaries, and research news stories for your research at: www.wileyauthors.com/eeo/promotion.

• If you would like to receive our 'Research Roundup', a monthly newsletter highlighting the cutting-edge research published in The Physiological Society's family of journals (The Journal of Physiology, Experimental Physiology, Physiological Reports, The Journal of Nutritional Physiology and The Journal of Precision Medicine: Health and Disease), please click this link, fill in your name and email address and select 'Research Roundup':
https://www.physoc.org/journals-and-media/membernews

EDITOR COMMENTS

Thank you for your careful revision in response to the reviewers' comments, and for submitting this excellent work to The Journal of Physiology. I am pleased to inform you that your manuscript has been accepted for publication. Congratulations on a fine study!

REFEREE COMMENTS

Referee #1:

The authors addressed the issues appropriately. No further comments.

Referee #2:

The authors have responded to the comments point by point and made appropriate revisions.

I have no further suggestions. Truly good work, thank you.
